# Dawn and Dusk Set States of the Circadian Oscillator in Sprouting Barley (*Hordeum vulgare*) Seedlings

**DOI:** 10.1371/journal.pone.0129781

**Published:** 2015-06-11

**Authors:** Weiwei Deng, Jenni Clausen, Scott Boden, Sandra N. Oliver, M. Cristina Casao, Brett Ford, Robert S. Anderssen, Ben Trevaskis

**Affiliations:** 1 CSIRO, Agriculture, GPO Box 1600, Canberra, ACT, 2601, Australia; 2 Max Planck Institute for Plant Breeding Research, Carl-von-Linné-Weg 10, Cologne, D50829, Germany; 3 CSIRO, Digital Productivity, GPO Box 664, Canberra, ACT 2601, Australia; Institute of Botany, Chinese Academy of Sciences, CHINA

## Abstract

The plant circadian clock is an internal timekeeper that coordinates biological processes with daily changes in the external environment. The transcript levels of clock genes, which oscillate to control circadian outputs, were examined during early seedling development in barley (*Hordeum vulgare*), a model for temperate cereal crops. Oscillations of clock gene transcript levels do not occur in barley seedlings grown in darkness or constant light but were observed with day-night cycles. A dark-to-light transition influenced transcript levels of some clock genes but triggered only weak oscillations of gene expression, whereas a light-to-dark transition triggered robust oscillations. Single light pulses of 6, 12 or 18 hours induced robust oscillations. The light-to-dark transition was the primary determinant of the timing of subsequent peaks of clock gene expression. After the light-to-dark transition the timing of peak transcript levels of clock gene also varied depending on the length of the preceding light pulse. Thus, a single photoperiod can trigger initiation of photoperiod-dependent circadian rhythms in barley seedlings. Photoperiod-specific rhythms of clock gene expression were observed in two week old barley plants. Changing the timing of dusk altered clock gene expression patterns within a single day, showing that alteration of circadian oscillator behaviour is amongst the most rapid molecular responses to changing photoperiod in barley. A barley *EARLY FLOWERING3* mutant, which exhibits rapid photoperiod–insensitive flowering behaviour, does not establish clock rhythms in response to a single photoperiod. The data presented show that dawn and dusk cues are important signals for setting the state of the circadian oscillator during early development of barley and that the circadian oscillator of barley exhibits photoperiod-dependent oscillation states.

## Introduction

Plants, like most other organisms, exhibit rhythmic fluctuations in gene expression and metabolism synchronised with day-night cycles. Many of these rhythms persist in constant conditions due to the action of an internal circadian clock [1,2]. Time-of-day dependent processes regulated by the clock are referred to as clock outputs. The timing of daily clock rhythms can adjust in response to external light or temperature cues to remain synchronised with the external environment; a process known as entrainment [3].

Genetic studies of model systems such as Arabidopsis (*Arabidopsis thaliana*) have identified components of the plant circadian clock. These include the MYB transcription factor genes *CIRCADIAN CLOCK ASSOCIATED1 (CCA1)* and *LATE ELONGATED HYPOCOTYL (LHY)* and a series of *PSEUDO RESPONSE REGULATOR (PRR)* genes including *TIMING OF CAB EXPRESSION 1 (TOC1)* [4–6]. Transcript levels for these genes oscillate with regular daily peaks and troughs. *CCA1* and *LHY* expression peaks in the morning, whereas *TOC1* expression peaks in the evening [7]. Other *PRR* genes are expressed with peaks staggered throughout the day [8]. Genetic and biochemical experiments have established that cross regulation occurs between clock genes and this is required to establish rhythmic gene expression patterns (reviewed in [9]). Current models state that *TOC1* represses *CCA1*, *LHY* and other *PRR* genes, which in turn down-regulate *TOC1* [10,11]. Mathematical modelling supports the idea that feedback loops generate rhythmic expression patterns of clock genes [12]. Other genes have been shown to influence the maintenance of circadian oscillations when plants are shifted from day-night cycles to constant light. These include *GIGANTEA (GI)* and *EARLY FLOWERING3 (ELF3)* [13–15]. These genes can also be considered as components of the plant circadian clock. Collectively circadian clock genes form a molecular mechanism that regulates daily rhythms of clock outputs.

As well as regulating daily gene expression rhythms, the circadian clock is thought to provide an internal timer that allows perception of changing daylength [16–18]. This is critical for photoperiod-dependent seasonal responses, such as daylength-induced flowering responses. A key component of the long-day flowering response of Arabidopsis is *CONSTANS (CO)*, a clock-regulated gene with a daily transcriptional rhythm that peaks in the late afternoon [18]. In long days the peak of *CO* expression coincides with light, which stabilises the CO protein [19]. CO protein then activates transcription of *FLOWERING LOCUS T* (*FT*) which encodes a mobile signal that is transported from leaves to the shoot apex to accelerate flowering [20]. Thus, in Arabidopsis, circadian regulation of *CO* underlies the seasonal flowering-response triggered by long days. Circadian rhythms also influence seasonal temperature responses, such as cold acclimation; the process whereby a period of chilling induces increased tolerance to freezing (see [21]).

The circadian clock plays a central role in fundamental biological processes that underlie agronomic performance of crop plants, so the functions of cereal clock genes are of critical interest to agriculture. Genes related to those that control circadian rhythms in Arabidopsis have been identified in cereal crops [22–26]. There is not a direct relationship between key clock genes in cereals versus those in Arabidopsis, however. *CCA1* and *LHY* have a single equivalent in rice, for example, and the *PRR* gene family has diverged independently in cereals versus Arabidopsis [23]. This divergence in clock gene sequences potentially underlies functional differences between the circadian clock of cereals and that of the dicot model Arabidopsis. Further research directly in cereals or related grass species is required to interpret how knowledge generated in model systems such as Arabidopsis might be relevant to cultivation of cereal crops.

Barley is a transformable diploid cereal that provides a useful model system for other temperate cereal crops, including wheat (*Triticum* spp.), oats (*Avena sativa*) and rye (*Secale cereale*), and also for a broader group of temperate pooid grasses. The circadian oscillator of barley appears to have similar properties and functions to that of Arabidopsis [24]. Transcript levels of barley circadian clock genes oscillate in day-night cycles and these oscillations continue in constant light/temperature conditions [24]. Mutations in genes that disrupt circadian clock function can alter seasonal flowering responses [25–27]. For example, loss-of-function mutations in the barley *ELF3* dampen circadian oscillations and confer a photoperiod-insensitive early-flowering phenotype [25,26]. These mutations have been used to breed barleys for regions with short growing seasons [25,26].

In this study we examine when oscillations of clock genes are first initiated in barley, an important crop and a model system for other temperate cereals and related grasses. Data are presented to show that oscillations of clock gene transcript levels do not occur in barley seedlings grown in constant conditions, but can be triggered by a single photoperiod. Evidence that dawn and dusk signals drive circadian oscillations is presented.

## Materials and Methods

### Growth conditions and plant material

Seeds were germinated on the surface of a 5 ml volume of 50% perlite:50% vermiculite saturated with water, plus 1.4 g/L of Thiram fungicide (Bayer Crop Science, www.bayercropscience.us) in 15 ml falcon tubes (Greiner, www.greinerbioone.com). Tubes were incubated at 20°C in Echotherm programmable temperature blocks (Torrey Pines Scientific Instruments, California, USA) in custom built fan ventilated chambers that were placed inside a cold room set at 4°C. The chambers (more details of construction can be provided on request) were designed to be light proof but were fitted with 40W incandescent lights to allow exposure to low intensity light (5 μmol m^-2^s^-1^). The temperature block was clad in a polystyrene jacket to limit heat dissipation and this was further covered in foil to limit light penetration (removed for light treatments). Seedlings were grown to a coleoptile length of 4 cm before the start of each experiment. Long and short day experiments (seedlings grown in different photoperiods for multiple days) were conducted in growth chambers (Conviron CMP6050) set at a constant temperature (20°C), with a light intensity of 250 μmol m^-2^s^-1^. For photoperiod shift experiments), plants were grown to the second leaf stage in growth chambers (see above) and whole plants, minus roots, were harvested. The vernalization-responsive winter barley cultivar Sonja (*HvVRN1*, *HvVRN2*, *PPD-H1*) used in the majority of experiments has been described previously, including detailed analyses of photoperiod-dependent flowering-responses [28]. The *HvELF3* (Mat.a8) mutant and the wildtype parent (cv. Bonus, *HvVRN1-1*, *ΔHvVRN2*, *ppd-H1*) have been described previously [25,26]. The isolation of the imbibed embryo samples has also been described previously [29].

### Gene expression analyses

RNA was extracted from individual barley seedlings (minus roots, which were removed at the base of the crown) using the method of Chang et al. [30]. Total RNA (5 μg) was reverse-transcribed with Super Script III reverse transcriptase (Invitrogen, www.invitrogen.com), according to manufacturer instructions. Quantitative Reverse Transcriptase PCR (qRT-PCR) was performed in a Rotorgene Q real-time PCR machine or a 7900HT Fast Real-Time PCR System (Applied Biosystems, http://www.appliedbiosystems.com) with SYBR green and Platinum Taq DNA polymerase (Invitrogen). The *ACTIN* gene was used as reference and relative transcript levels were calculated with the ΔΔCt method, factoring in primer amplification efficiencies as described previously [31]. Primer sequences are described in S1 Table. Barley clock gene sequences have been described previously by Campoli et al., [24]. The sequences of *HvVRN2*, *HvPPD1*, *HvCO1*, *HvGRP7* and *HvLHCII* have also been reported previously [32–34].

## Results

### Clock genes do not oscillate in seedlings germinated in constant conditions

**Fig 1:**
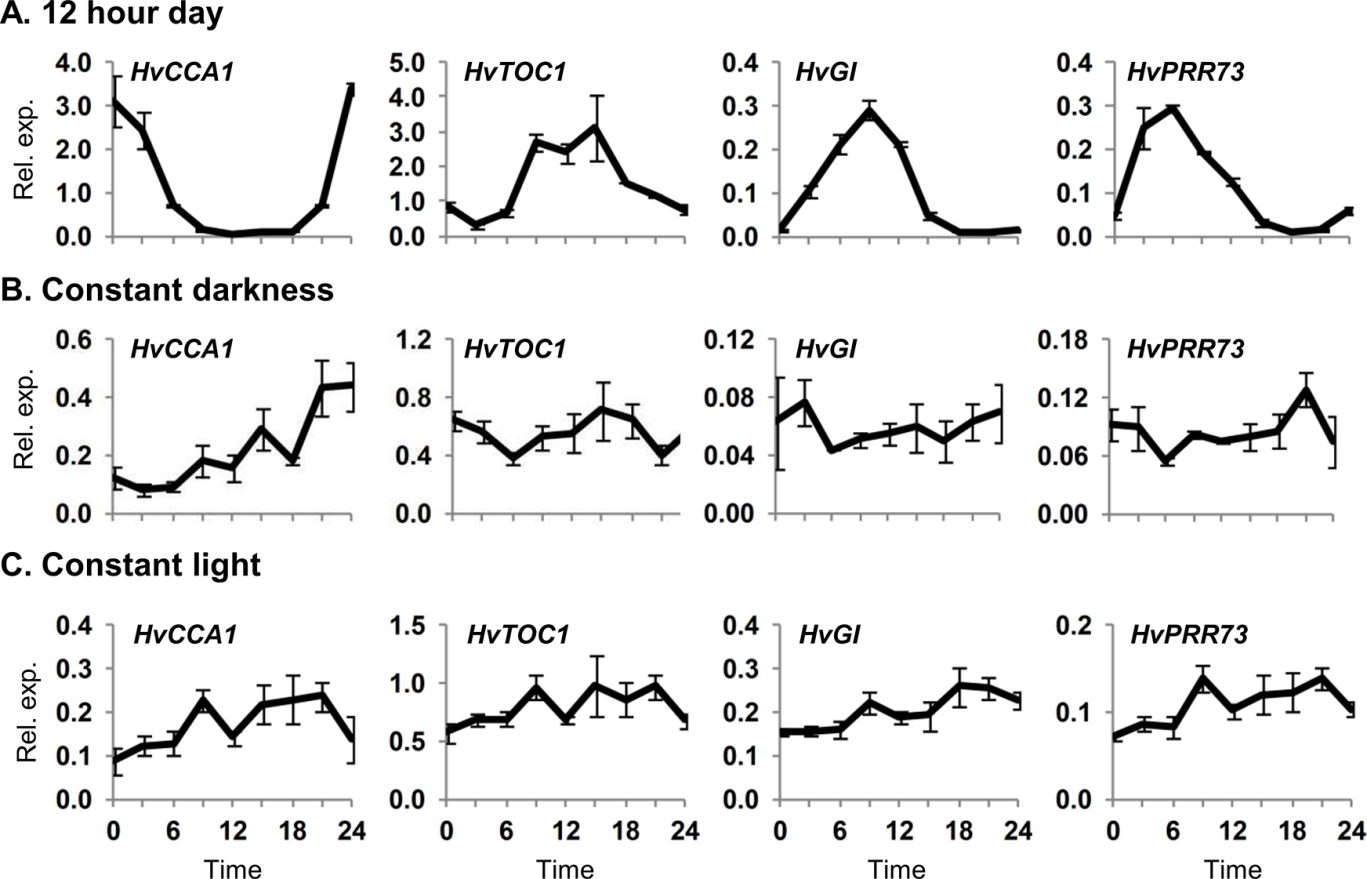
Expression of clock genes assayed in barley seedlings grown in different light regimes. Clock gene expression assayed by quantitative reverse transcriptase PCR (qRT-PCR) in 5 day old barley seedlings (cv. Sonja) germinated and grown in constant temperature conditions (20°C) with (A) 12 hour day-night cycles with light from hours 0 to 12, (B) constant darkness or (C) constant light. Average expression in RNA extracted from individual seedlings (3 biological repeats) is shown relative to *ACTIN* (Rel. exp.), error bars show standard error. Horizontal axis labels indicate the time (hours) relative to when the first sample was harvested. Samples were harvested directly from the described conditions (i.e. without free running conditions for the day-night cycle samples).

Clock gene transcript levels were first assayed in 5 day old barley seedlings grown in 12 hour day-night cycles. Rhythmic oscillations of transcript levels were observed for several clock genes including *HvCCA1*, *HvTOC1*, *HvGI*, *HvPRR73*, *HvPRR59* and *HvPRR95* (Fig 1A, S1 Fig), similar to previous reports (e.g. [24]). A number of clock-regulated genes also showed rhythmic expression, consistent with the observed clock gene oscillations; *PHOTOPERIOD1 (HvPPD1*), *VERNALIZATION2* (*HvVRN2*), a barley *CO* orthologue (*HvCO1*), a glycine-rich protein (*HvGRP7*, also known as *CIRCADIAN CLOCK REGULATED2*, *CCR2)* and chlorophyll AB binding proteins (*HvCAB1*, *HvLHCII*) (S2 Fig).

Some studies suggest that imbition of seeds drives the establishment of circadian oscillations in Arabidopsis [35,36]. Clock gene expression was assayed in barley embryos dissected from seeds at various timepoints during imbition in darkness. No oscillations in clock gene transcript levels were observed in embryos during the first day of imbition, with transcript levels of most clock genes remaining similar to those at the initial timepoint in dry embryos (S3 Fig).

Gene expression patterns were then examined in 5 day old seedlings germinated and grown without day-night cycles to examine whether rhythms initiate during early seedling development in the absence of external light/dark cues. Clock gene transcript levels did not show rhythmic oscillations in darkness and were generally similar to the midpoint expression value across the time course examined (Fig 1B). The exception was *CCA1* expression, which gradually increased over the time-course examined (Fig 1B). The overall state of the circadian clock in dark grown seedlings did not resemble the oscillating state seen in day-night cycles and clock-regulated genes did not show rhythmic oscillations (S1 Fig). Similar results were obtained in multiple independent experiments with dark grown seedlings.

### Dawn and dusk signals induce circadian oscillations in barley

**Fig 2:**
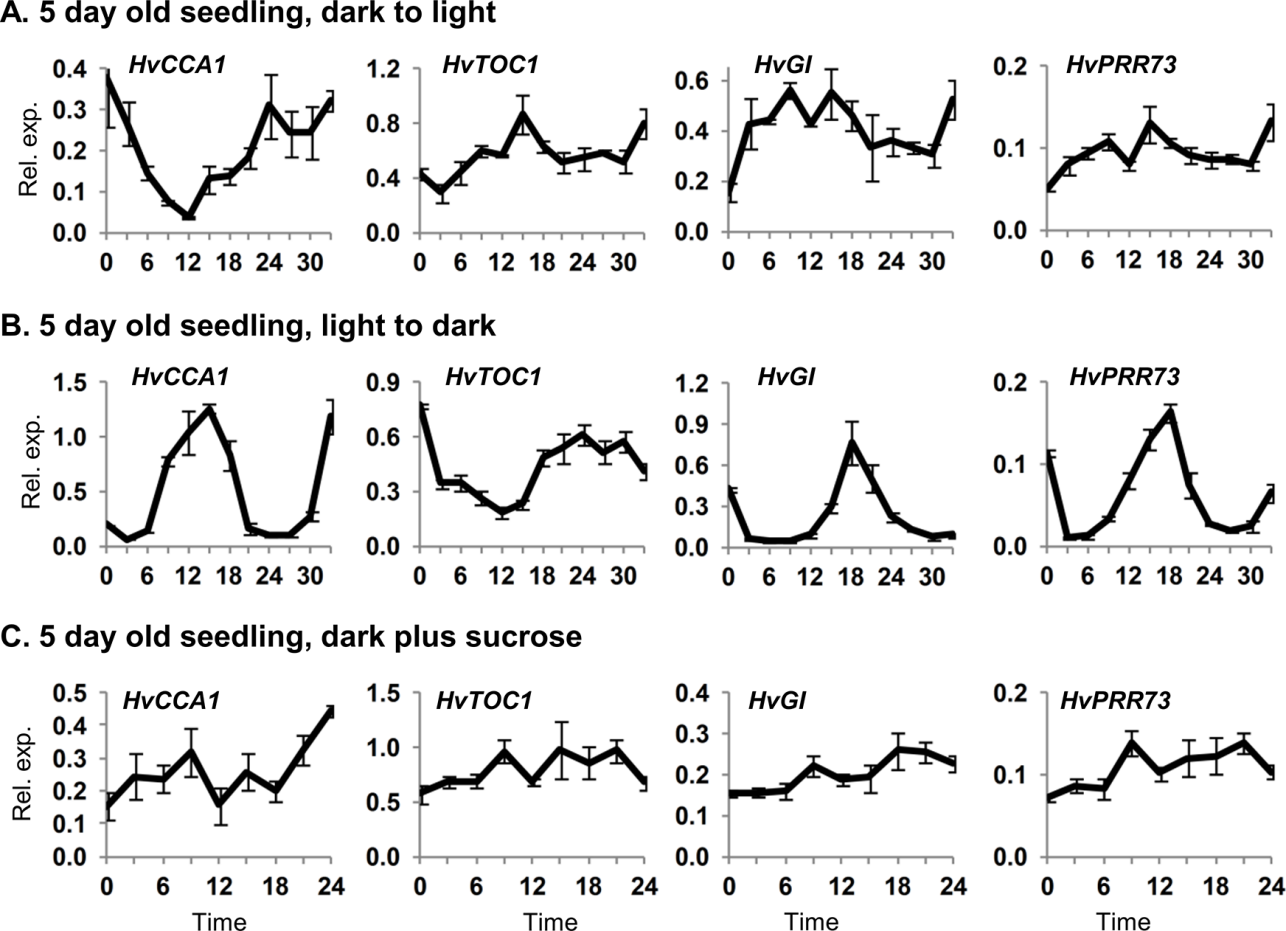
Clock gene expression after light/dark transitions or with exogenous sucrose. Gene expression, assayed by qRT-PCR, in 5 day old barley seedlings (cv. Sonja) germinated and grown in: (A) constant darkness then shifted to light, (B) constant light then shifted to darkness or (C) constant darkness plus 2% sucrose. RNA was extracted from 3 biological repeats. Average expression is shown relative to *ACTIN* (Rel. exp.), error bars show standard error. Horizontal axis labels indicate the time (hours) relative to when the first sample was harvested and treatments began.

Light can activate circadian oscillations in Arabidopsis [37–39]. The expression behaviour of clock genes in barley seedlings grown in constant light was similar to constant darkness (Fig 1C), though *HvGI* transcript levels were elevated in constant light compared to darkness (S4 Fig). A single transition from darkness to constant light triggered changes in clock gene transcript levels in barley seedlings. Initially expression of *HvGI* and *HvPRR73* increased whereas expression of *HvCCA1* decreased (Fig 2A). Analysis of gene expression over a narrower time course shows that *HvGI* and *HvPRR95* transcript levels increase rapidly in response to light (S5 Fig). Beyond these initial gene expression changes there were only weak fluctuations of clock gene transcript levels (Fig 2A). Seedlings germinated in constant light were then shifted to darkness. Initially transcript levels of all clock genes declined. Thereafter, there were robust oscillations in clock gene transcript levels (Fig 2B). Clock-regulated genes also showed strong responses to a light-to-dark transition (S6 Fig). Sucrose, which influences circadian rhythms in Arabidopsis [40–41], did not influence clock gene expression when added to barley seedlings grown in constant darkness (Fig 2C).

### A single photoperiod activates circadian oscillations in barley

**Fig 3:**
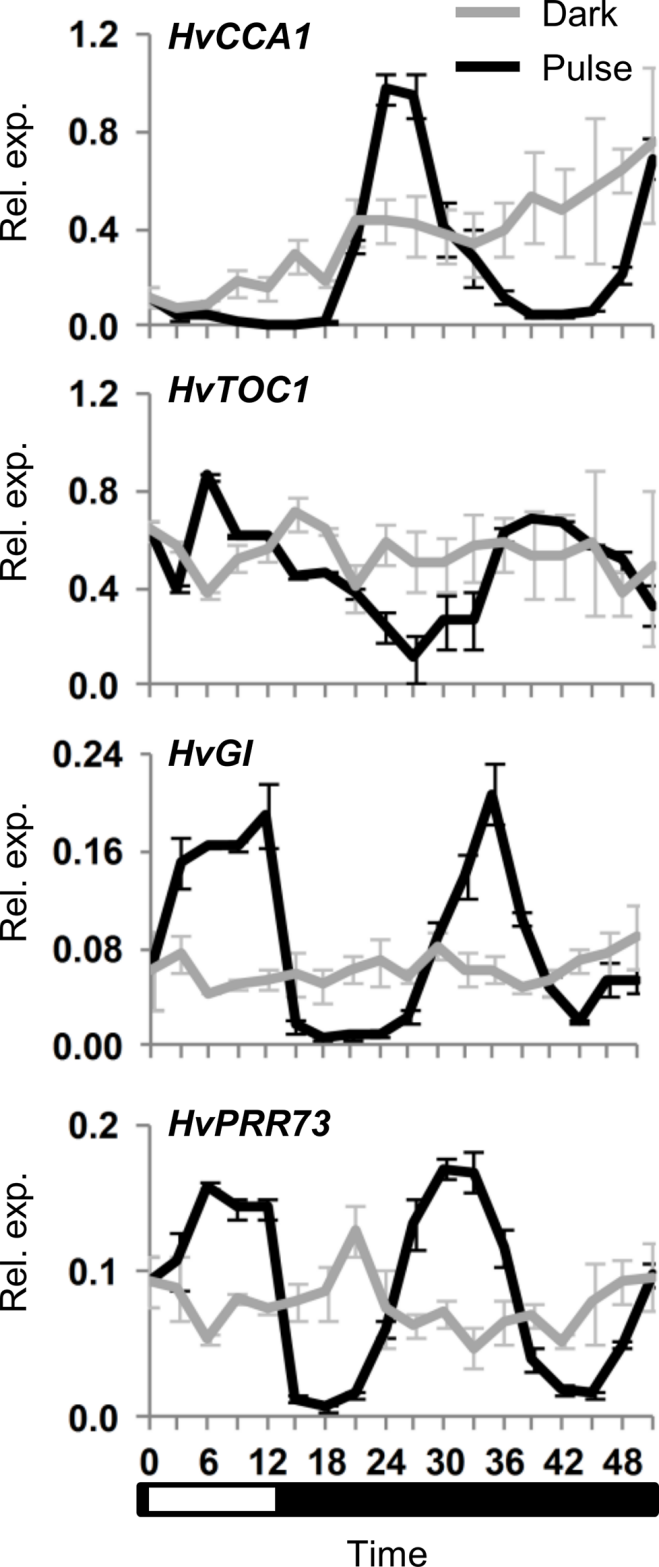
Initiation of circadian oscillations following a single light pulse. Clock gene expression, assayed by qRT-PCR, in 5 day old barley seedlings (cv. Sonja) germinated and grown in constant darkness then exposed to a 12 hour light pulse (black line) versus control plants maintained in darkness (grey line). RNA was extracted from 3 biological repeats. Average expression is shown relative to *ACTIN* (Rel. exp.), error bars show standard error. Horizontal axis labels indicate the time (hours) relative to when the first sample was harvested. The white and black bar indicates duration of the light period relative to sampling timepoints (white corresponds to the light period).

Plants were exposed to a single 12 hour light pulse. The initial response to light was similar to that described previously, with increased *HvGI* and *HvPRR73* expression (Fig 3). Expression of these genes remained elevated until the onset of darkness then declined. Thereafter, transcript levels of clock genes continued to fluctuate in constant darkness (Fig 3). *HvGI* transcript levels increased again 24 hours after the exposure to light began, then decreased 24 hours after lights were switched off, for example (Fig 3). The second increase of *HvPRR73* began within 18–21 hours, preceding increased *HvGI* expression (Fig 3). Oscillations of *HvGI* transcript levels continued for three days following a single 12 hour light pulse, though the amplitude of oscillations declined during this period (S7 Fig).

### The response of barley clock genes to single photoperiods of different durations

**Fig 4:**
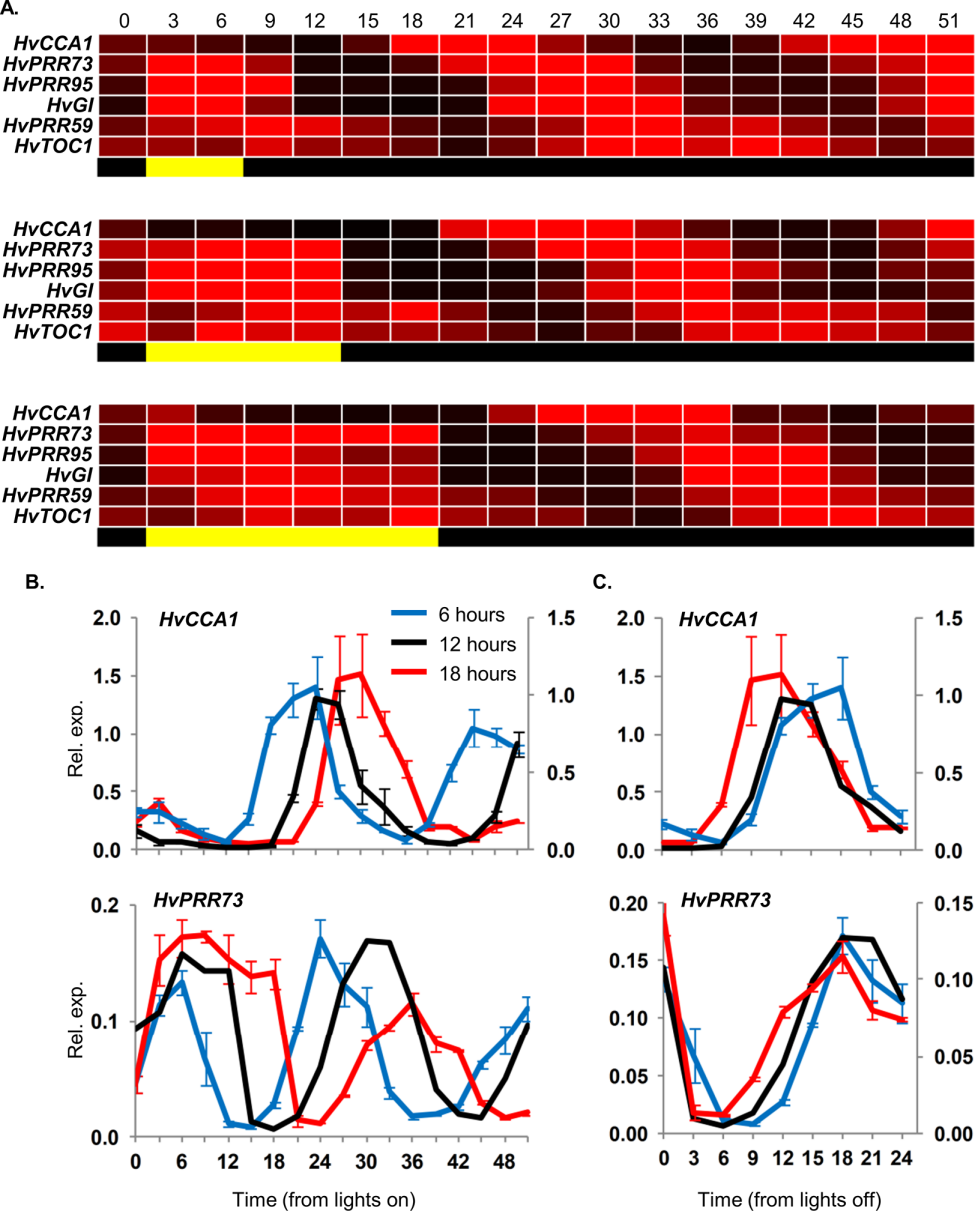
Initiation of circadian oscillations following single light pulses of different durations. (A) Heat map of clock gene expression, assayed by qRT-PCR, in 5 day old barley seedlings (cv. Sonja) germinated and grown in constant darkness then exposed to a single light pulse of 6, 12 or 18 hours. Expression is presented ranged from 0 (black) to 2-fold increase (red) in transcript levels, relative to median expression of that gene across all timepoints in the specific experiment. Light treatments are represented by yellow and black bars beneath each heatmap. (B) Transcript levels of *HvCCA1* and *HvPRR73* in the same experiment with single light pulses of 6 (blue line), 12 (black line) or 18 hours (red line), plotted from the start of the light pulse treatments. (C) Transcript levels of *HvCCA1* and *HvPRR73* from the end of the light pulses (dusk). RNA was extracted from 3 biological repeats. Average expression is shown relative to *ACTIN* (Rel. exp.), error bars show standard error. Relative transcript levels varied between treatments so two y-axes were used to allow easy comparison of overall rhythms; the 18 hour treatment is shown on the secondary axis (right-hand side). Horizontal axis labels indicate the time (hours) relative to when the first sample was harvested.

Barley seedlings grown in darkness were exposed to single light pulses of 6 or 18 hours duration to examine how different photoperiods might influence clock initiation. The initial responses to light were similar to those seen in the 12 hour light pulse treatment (Fig 4). Oscillations did not begin until the end of the light period and consequently the timings of subsequent fluctuations in clock gene transcript levels were determined primarily by the light-to-dark transition (Fig 4B versus 4C). There were, however, distinct differences in the timing of oscillations after the light-to-dark transition for the different photoperiod treatments. For example, when transcript levels of *HvCCA1* are plotted relative to the end of the light period, peak expression occurs earlier after an 18 hour light pulse than for the other light pulse durations (Fig 4C). So, while oscillations begin at the end of the light period, the duration of the light period does influence subsequent oscillation patterns. A single long photoperiod induced expression of *HvVRN2* (S8 Fig), a gene known to be expressed specifically in long days [31].

### Expression of barley clock genes in seedlings grown under different photoperiods

**Fig 5:**
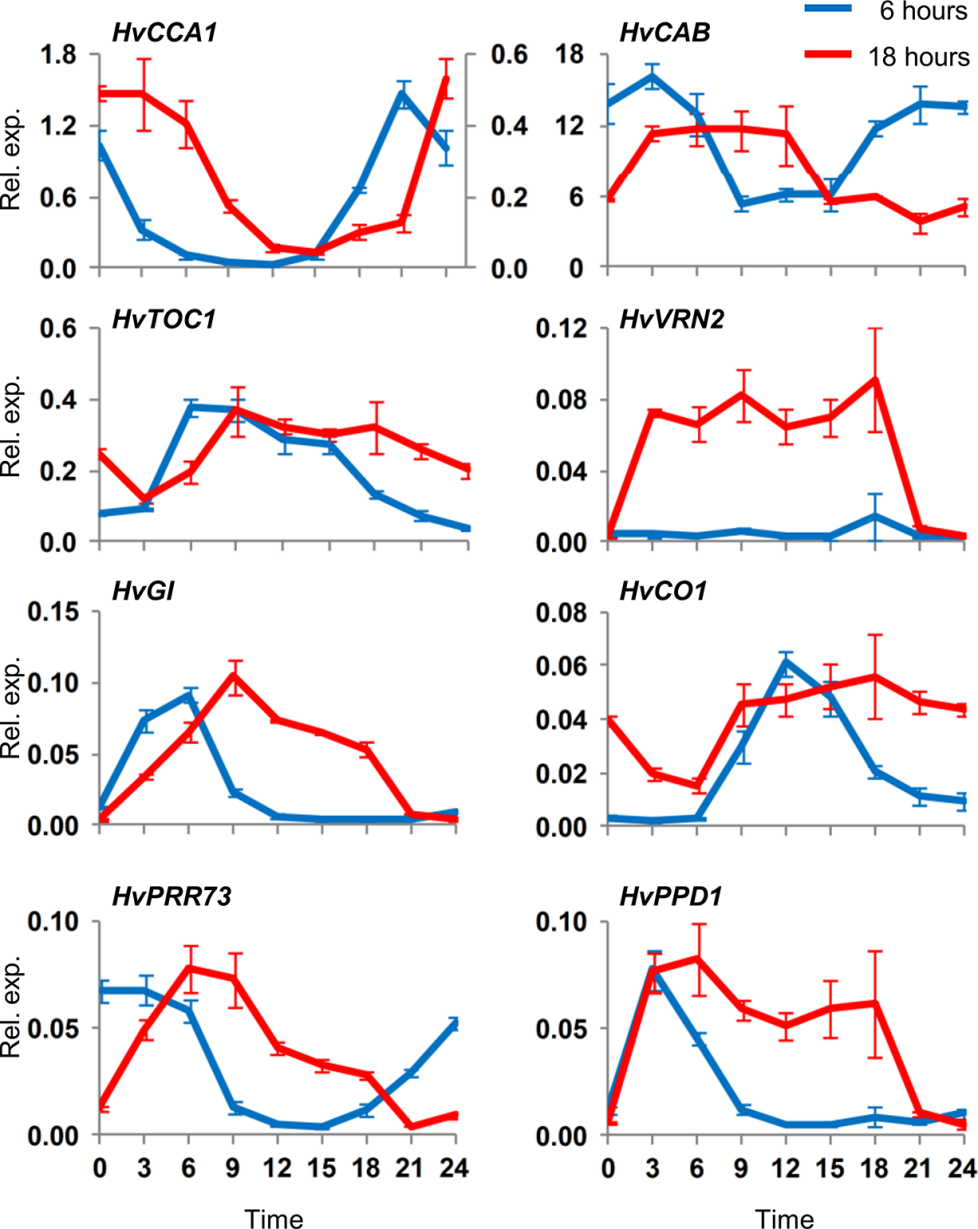
Photoperiod dependence of circadian oscillations in barley seedlings. Transcript levels of clock or clock-regulated genes, assayed by qRT-PCR, in 5 day old barley seedlings (cv. Sonja) germinated and grown in 6 (blue line) or 18 hour daylengths (red line). Dawn was synchronised between the two photoperiod treatments. RNA was extracted from 3 biological repeats. Average expression is shown relative to *ACTIN* (Rel. exp.), error bars show standard error. Horizontal axis labels indicate the time (hours) relative to dawn when the first sample was harvested.

Seedlings were grown in short (6 hr) or long (18 hr) days to examine clock gene expression patterns following multiple day-night cycles with different photoperiods. Clear differences in the timing of peak/trough transcript levels of *HvCCA1*, *HvTOC1*, *HvGI* and *HvPRR73* were observed between the short versus long-day treatments (Fig 5). Daylength specific gene expression patterns were also observed for clock-regulated genes (Fig 5). In both short and long days, expression of *HvGI* was high in the light period and declined at dusk, remaining low throughout the night (Fig 5). Expression of clock genes in short and long days was then compared with expression patterns induced by a single photoperiod; specifically gene expression in darkness during the 24 hours post light pulse. A single photoperiod of 6 hours was sufficient to establish internal rhythms similar to those seen with multiple day-night cycles of equivalent photoperiod. A single 18 hour photoperiod was not able to generate the same rhythm as that observed with multiple day-night cycles (S9 Fig).

### Rapid adjustment of circadian oscillations in response to altered photoperiod

**Fig 6:**
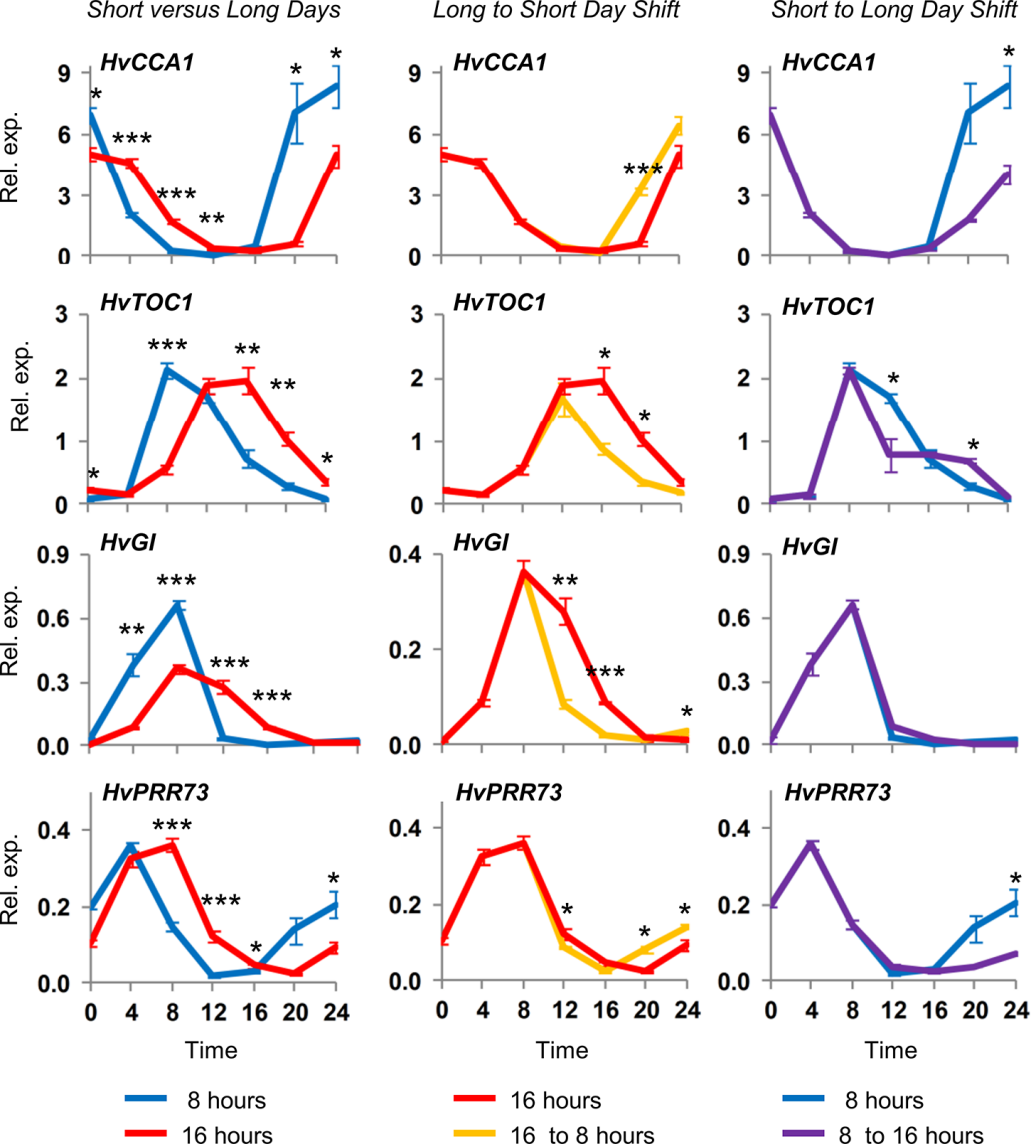
Dynamic response of circadian oscillations to daylength shifts. Barley seedlings (cv. Sonja) were germinated and grown in 8 hour or 16 hour days, for 14 days (second leaf stage), with dawn synchronous in the two daylengths. On the 14^th^ day plants were shifted from 8 to 16 hour days and *vice versa*. Shifts occurred 8 hours after dawn shortly before the onset of darkness in the short-day condition. Gene expression was then assayed by qRT-PCR, normalized to *ACTIN* with 3 biological repeats, in the plants shifted to different daylengths and in control plants maintained in the same daylength. The 8 hour day treatment (blue line) is compared to 16 hours (red line) at the left hand side. The 16 hour day treatment is compared to plants shifted from 16 to 8 hour daylength (long to short days, orange line), centre panel. The 8 hour daylength is compared to seedlings shifted to 16 hours (short to long days, purple line) on the right hand side. Error bars show standard error. * indicates Student’s T-test P<0.05, **P<0.01, ***P<0.001 between treatments for the relevant timepoint.

Seedlings were grown in 8 or 16 hour photoperiods, contrasting daylength conditions commonly used to assess the impact of daylength on cereal physiology and development, until plants reached the second leaf stage. Clear differences in clock gene expression patterns were seen between 8 versus 16 hour photoperiods (Fig 6). Then, reciprocal shifts were made between the two growth conditions. Irrespective of the direction of the transfer, a shift of photoperiod altered the expression patterns of clock genes (Fig 6). The only exception was *HvGI*, which did not respond to a longer day, despite showing a strong response to a shorter day (Fig 6).

### *EARLY FLOWERING3* is required for the single-photoperiod response of the circadian oscillator in barley seedlings

**Fig 7:**
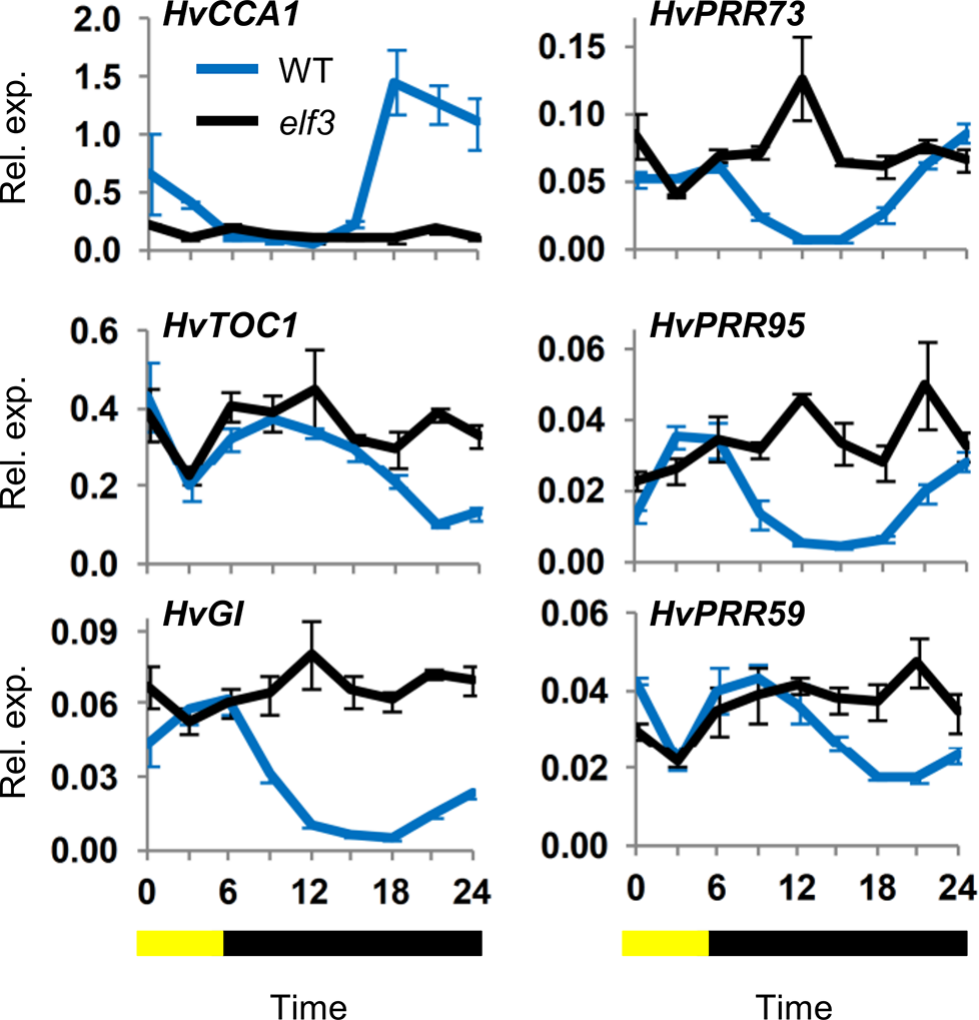
*ELF3* function is required for induction of transcriptional oscillations by a single light pulse. Transcript levels of clock genes assayed qRT-PCR normalized to *ACTIN* (average of 3 biological repeats) in 5 day old barley seedlings (cv. Bonus) that were germinated and grown in constant darkness then exposed to a single light pulse of 6 hours (blue line). Expression is compared to a *HvELF3* loss-of-function mutant (black line) isolated in the same genetic background. Error bars show standard error. The yellow and black bar indicates duration of light period relative to sampling timepoints.

Barleys that lack *HvELF3* gene function show disrupted circadian rhythms and are photoperiod insensitive, flowering early irrespective of daylength [25,26]. To examine whether the *HvELF3* gene is required for the initiation of circadian rhythms in barley seedlings, the response of the circadian oscillator to a single photoperiod was examined in a *HvELF3* loss-of-function mutant versus the wildtype parent (Fig 7, S10 Fig). When exposed to a single short photoperiod (6 hours) the changes in clock gene expression observed in the wildtype parent line (Fig 7) were similar to those described earlier (Fig 3). The same responses were not observed in the *HvELF3* mutant, where clock gene expression remained arrhythmic (Fig 7). Notably, the light-to-dark transition (dusk) did not influence clock gene expression in the *HvELF3* loss of function mutant. *HvGI* was expressed at high basal levels throughout the time course in the *HvELF3* mutant, resembling the expression pattern observed in seedlings grown in constant light (S4 Fig versus S10 Fig).

## Discussion

Clock gene transcript levels do not oscillate rhythmically in barley seedlings grown without day-night cycles (Fig 1, S1 Fig). One explanation for this observation is that transcript levels do not oscillate for these genes. An alternative explanation is that oscillations occur asynchronously between individual cells, averaging to arrhythmic expression in whole seedlings (see [42]). Regardless of the underlying basis, the oscillating state is functionally different to the non-oscillating state, since *HvGI* shows a gated response to light in seedlings with induced clock rhythms but a uniform response to light throughout a 24 hour period in seedlings grown in constant darkness (S11 Fig). Furthermore, the response of some clock genes to the initial light response differs to that seen at dawn in plants grown in day-night cycles. *HvPRR73*, for example, shows a peak in transcript levels after 3–6 hours after dark-grown seedlings are exposed to light (Fig4A), but this is not seen in day-night cycles where the peak of expression for this gene precedes dawn.

Growing barley seedlings in constant light altered transcript levels of some clock genes relative to constant darkness but did not induce circadian rhythms (S4 Fig). Thus, light *per se* does not trigger circadian oscillations in barley seedlings. Similarly, a single transition from dark to light triggered rapid changes in transcript levels of some barley clock genes, *HvGI* and *HvPRR95* in particular, but did not induce robust rhythmic oscillations (Fig 2A, Fig 4A, S5 Fig). Instead, exposure to light followed by transition to darkness appears to be a more effective treatment for activating oscillations. This suggests that both dawn and dusk cues, together comprising a single photoperiod, are important signals that regulate the initiation of circadian oscillations. Dusk (light to dark) seems to be a particularly important cue for the initiation of circadian oscillations. Two lines of experimental evidence support this conclusion. (a) A single transition from constant light to dark triggered rhythmic expression of clock genes (Fig 2B), similar to those seen in day-night cycles. (b) When seedlings were exposed to a single light pulse clock gene expression initially showed a light dependent phase, where *HvGI* and *HvPRR73* transcript levels increased, but oscillating transcript levels were not observed until after the end of the light pulse (a light-to-dark transition) (Fig 3).

The duration of light prior to a light-to-dark transition influences the timing subsequent circadian rhythms (Fig 4B), so the initial rhythms of the barley circadian oscillator appear to be photoperiod dependent. Consistent with this hypothesis, circadian oscillations in barley plants are photoperiod dependent and changes in photoperiod trigger rapid changes in the timing of circadian oscillations (Fig 5, Fig 6). Photoperiod-specific states of the circadian oscillator were associated with daylength-specific expression patterns for photoperiod-response regulators, including *HvVRN2*, *HvCO1* and *HvPPD1* (Fig 5). Interestingly, seedlings that lacked pre-existing circadian oscillations show daylength-specific responses to a single photoperiod, with induction of *HvVRN2* occurring only in response to a long photoperiod pulse (S8 Fig). Thus, single photoperiods can simultaneously induce both photoperiod-dependent clock states and photoperiod-specific expression of a daylength response regulator. Rapid activation of daylength-dependent circadian rhythms might allow seedlings to quickly establish daylength responses appropriate for the seasonal conditions encountered upon germination. Of course, temperature fluctuations are another cue likely to affect the state of the circadian oscillator and this will be a topic for future studies.

*ELF3* function seems to be critical for the response to light-to-dark (dusk), which had little impact on clock gene expression in a *HvELF3* loss-of-function mutant (Fig 7). For some clock genes there was also a reduced response to the dark-to-light transition, e.g. *HvGI*, *HvPRR95* (Fig 7). The ELF3 protein is thought to function in a multimeric “evening complex”, with ELF4 and LUX, which represses transcription of other clock genes [15,39,43–46]. Constitutive de-repression of light-induced components of the circadian oscillator, such as *HvGI* and *HvPRR95*, might account for the reduced sensitivity of *HvELF3* to dawn and dusk cues. The *HvELF3* loss-of-function mutant has elevated *HvGI* expression in constant darkness, consistent with this hypothesis (S10 Fig). In terms of photoperiod responses of evening complex genes in a wildtype barley background, *HvLUX1* showed similar expression patterns to other clock genes in seedlings grown in different photoperiod treatments, with no rhythms in constant darkness and photoperiod-dependent expression in day-night cycles (S12 Fig). *HvELF3* did not show pronounced rhythms in constant temperature conditions and was not light responsive (S12 Fig).

Detailed analyses of clock gene activity during early seedling development have been conducted in Arabidopsis. Some studies suggest that oscillations do not occur in Arabidopsis seedlings germinated in constant darkness [37–39]. Other studies suggest that imbition triggers initiation of circadian oscillations [35–36]. Data from a recent study suggest that oscillations of *TOC1* might be induced in Arabidopsis seeds during imbition with light exposure but decline thereafter [47]. It is possible, therefore, that activation of Arabidopsis clock rhythms during imbition might be caused by exposure to light, as opposed to imbition *per se*. Studies that detected non-oscillating clock gene expression in Arabidopsis seedlings germinated in darkness found that a shift to constant light induced a peak of *CCA1* expression within 12 hours, before any light-to-dark transition [37–39]. This contrasts with barley, where *HvCCA1* expression declines with the onset of light and only increased above basal levels after a light-to-dark transition. Whether the initiation of clock rhythms in Arabidopsis displays any photoperiod specificity is not currently known. Photoperiod dependent clock states have been demonstrated in Arabidopsis plants [48] but shifting the timing of dusk has less impact on the Arabidopsis circadian oscillator [49] compared to barley (this study). Increased responsiveness to dusk cues is potentially a point of difference between the circadian oscillators of barley and Arabidopsis.

## Conclusions

Most studies of the plant circadian oscillator have examined the maintenance of circadian oscillations when plants are shifted from day-night cycles to constant conditions. This study used the alternative approach of examining the establishment of circadian oscillations from a non-oscillating state, which allows the influence of external cues on clock gene expression to be uncoupled from feedback caused by pre-existing oscillations. Using this approach it was possible to demonstrate an inherent photoperiod sensitivity of the barley circadian oscillator and to provide new insights into the potential basis for daylength insensitivity in a barley clock mutant. A key question for further research will be whether the shifting states of the circadian oscillator mediate photoperiod responses in plants, as opposed to merely providing an internal reference timer.

## Supporting Information

S1 Fig Overview of clock gene expression patterns in different treatments. Heatmaps summarizing expression patterns of clock genes in barley embryos or 5 day old seedlings in different light regimes, at different timepoints (hours) after the start of each experiment. Average expression, assayed by qRT-PCR normalized to *ACTIN* (3 biological repeats), is presented ranged from 0 (black) to 2-fold increase (red) relative to median expression of that gene across all timepoints in the specific experiment. Horizontal axis labels indicate the time (hours) relative to when the first sample was harvested. Coloured bars underneath each heatmap indicate light conditions for each experiment across the timepoints examined.
(TIF)

S2 Fig Expression of clock-regulated genes in barley seedlings grown in 12 hour day-night cycles. Gene expression patterns for clock-regulated genes, assayed by qRT-PCR, in 5 day old barley seedlings (cv. Sonja) germinated and grown in 12 hour day-night cycles. Average expression from 3 biological repeats is shown relative to *ACTIN* (Rel. exp.), error bars show standard error. Horizontal axis labels indicate the time (hours) relative to when the first sample was harvested.
(TIF)

S3 Fig Expression of clock genes in barley embryos during imbition. Gene expression, assayed by qRT-PCR, in barley embryos (cv. Betzes) isolated from dry seeds and at various timepoints after imbition. Detailed descriptions of morphological changes of embryos during the germination process during the time span have been presented previously by Barrero-Sanchez et al. [29]. Average expression is shown relative to *GAPDH* (Rel. exp.), error bars show standard error. Horizontal axis labels indicate the time (hours) relative to when the first sample was harvested. Similar results were obtained using *ACTIN* as a reference gene.
(TIF)

S4 Fig Gene expression of clock or clock-regulated genes in constant light versus constant darkness. Gene expression, assayed by qRT-PCR, in 5 day old barley seedlings (cv. Sonja) that were germinated and grown in constant darkness (black) or constant light (yellow). Average expression (3 biological repeats) is shown relative to *ACTIN* (Rel. exp.), error bars show standard error. Horizontal axis labels indicate the time (hours) relative to when the first sample was harvested.
(TIF)

S5 Fig Short-term responses of barley clock genes to light. Barley seedlings (5 days old, cv. Sonja) were shifted from constant darkness to light and harvested at different timepoints from 0 to 3 hours. Gene expression was assayed by qRT-PCR. Data are presented for: *HvCCA1*, *HvTOC1*, *HvGI*, *HvPRR73*, *HvPRR59* and *HvPRR95*. Expression is shown relative to *ACTIN* (Rel. exp.). Each data point is a mean of 3 biological repeats, error bars show standard error. Horizontal axis labels indicate time (hours) from the beginning of light exposure. * indicates Student’s T-test P<0.05, **P<0.01, ***P<0.001 versus the initial timepoint.
(TIF)

S6 Fig Expression of clock-regulated genes in barley seedlings shifted from constant light to darkness or *vice versa*. Gene expression, assayed by qRT-PCR, in 5 day old barley seedlings (cv. Sonja) germinated and grown in: constant darkness then shifted to light (left), or constant light the shifted to darkness (right). RNA was extracted from 3 biological repeats. Average expression is shown relative to *ACTIN* (Rel. exp.), error bars show standard error. Horizontal axis labels indicate the time (hours) relative to when the first sample was harvested and treatments began.
(TIF)

S7 Fig Peak versus trough expression of *HvGI* during a 5 day period from the time when seedlings were exposed to a single 12 hour light pulse. Barley seedlings (5 days old, cv. Sonja) were grown in constant darkness and then exposed to a 12 hour light pulse. Samples (2 biological repeats) were then collected at predicted times of peak (12 hours, then 36, 60 etc.) or trough expression (18 hours, 42, 66 etc), chosen on the basis of expression during the first 48 hours of the experiment, assayed at 3 hour intervals. Gene expression was assayed by qRT-PCR. Average expression is shown relative to *ACTIN* (Rel. exp.), error bars show range. Horizontal axis labels indicate the time (hours) relative to when the first sample was harvested.
(TIF)

S8 Fig Photoperiod response of *HvVRN2* to a single light pulse. Average transcript levels of *HvVRN2*, relative to *ACTIN*, in barley seedlings grown in darkness and then exposed to a single light pulse of 6 (blue line) or 18 hours (red line). Expression (3 biological repeats) was assayed by qRT-PCR, error bars show standard error. Horizontal axis labels indicate the time (hours) relative to when the first sample was harvested.
(TIF)

S9 Fig Comparison of circadian oscillations after/in single versus multiple photoperiods. Clock gene expression in short (6 hour light) or long days (18 hours light) versus the second day after a single photoperiod pulse of 6 or 18 hours. RNA was extracted from 3 biological repeats. Average expression is shown relative to *ACTIN* (Rel. exp.), error bars show standard error. Horizontal axis labels indicate the time (hours) relative to when the first sample was harvested. The transcript levels of some genes vary between treatments and so are plotted on different scales (y-axis) to allow easier comparison of overall expression patterns (i.e. timing of oscillations). In each case, the multiple photoperiods treatment is plotted on the primary (left) axis, the single light pulse on the secondary (right-hand).
(TIF)

S10 Fig Clock gene expression in wildtype versus *ELF3* loss-of-function mutant in constant darkness. Transcript levels of clock genes assayed qRT-PCR and normalized to *ACTIN* (3 biological repeats) in 5 day old barley seedlings (cv. Bonus) that were germinated and grown in constant darkness compared to a *HvELF3* loss-of-function mutant (black line). Error bars show standard error.
(TIF)

S11 Fig Acute induction of *HvGI* in dark grown seedlings, versus one day after a 12 hour photoperiod. Barley seedlings (5 days old, cv. Sonja) were maintained in constant darkness or exposed to a single 12 hour photoperiod. The next day, beginning at 24 hours after the onset of light, seedlings were exposed to 3 hours of light (grey bars) or maintained in darkness (black bars). Expression is shown relative to *ACTIN* (Rel. exp.). Average of 3 biological repeats, error bars show standard error. * indicates Student’s T-test P<0.05, NS is non-significant. Horizontal axis labels indicate the time of day (hours) when the light treatment began. The initial timepoint (0) is 24 hours after the start of the 12 hour light pulse.
(TIF)

S12 Fig Expression of *HvELF3* and *HvLUX1* in different photoperiod treatments. (A) Transcript levels of *HvELF3* in 5 day old seedlings (cv. Sonja) grown in constant darkness versus constant darkness followed by a single 12 hour light treatment, which started on the 5^th^ day. (B). Transcript levels of *HvLUX1* in constant darkness versus single 12 hour light treatment. (C) Transcript levels of *HvELF3* in 5 day old seedlings that were grown in 6, 12 or 18 hour photoperiods. (D) Transcript levels of *HvLUX1* in 5 day old seedlings that were grown in 6, 12 or 18 hour photoperiods. The 6 hour photoperiod is plotted on the secondary axis. Expression was assayed by qRT-PCR. Average expression is shown relative to *ACTIN* (Rel. exp.), error bars show standard error. Horizontal axis labels indicate the time (hours) relative to when the first sample was harvested.
(TIF)

S1 Table Primers used in for qRT-PCR analysis in this study. (DOCX)
