# Correction: Dawn and Dusk Set States of the Circadian Oscillator in Sprouting Barley (*Hordeum vulgare*) Seedlings

**DOI:** 10.1371/journal.pone.0138255

**Published:** 2015-09-11

**Authors:** Weiwei Deng, Jenni Clausen, Scott Boden, Sandra N. Oliver, M. Cristina Casao, Brett Ford, Robert S. Anderssen, Ben Trevaskis

There is an error in Fig 2C. Specifically, the panels for Fig 2C showing expression data for HvTOC1, HvGI and HvPRR73 were duplicates of panels from Fig 1C. The duplication of these images occurred during figure preparation and was an error made by the corresponding author (B. Trevaskis). The correction does not alter the conclusions of the study. The authors apologise for this error and thank the diligent reader who brought this to our attention.

Please see the complete, correct Fig 2 here.

**Fig 2:**
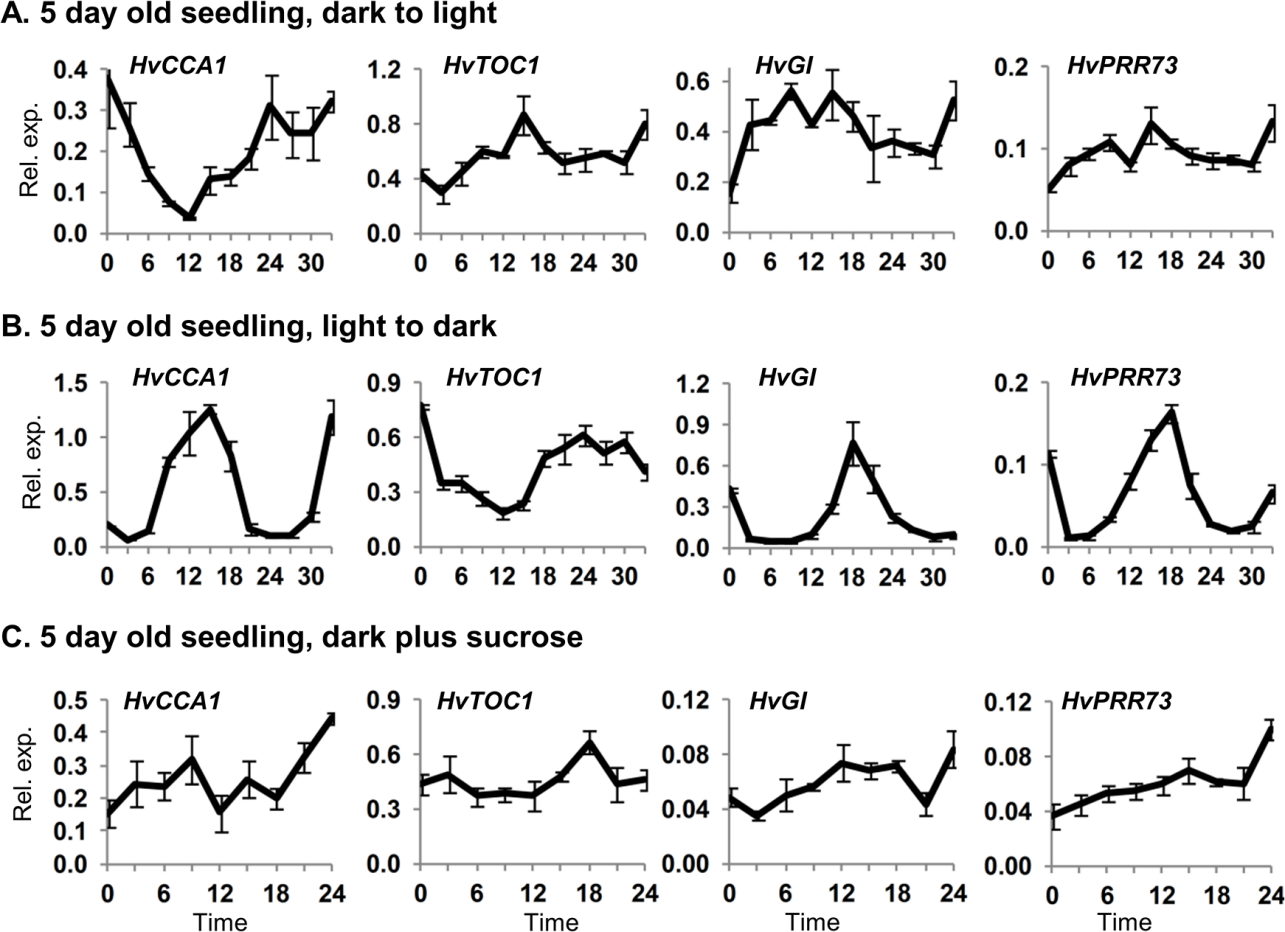
Clock gene expression after light/dark transitions or with exogenous sucrose. Gene expression, assayed by qRT-PCR, in 5 day old barley seedlings (cv. Sonja) germinated and grown in: (A) constant darkness then shifted to light, (B) constant light then shifted to darkness or (C) constant darkness plus 2% sucrose. RNA was extracted from 3 biological repeats. Average expression is shown relative to *ACTIN* (Rel. exp.), error bars show standard error. Horizontal axis labels indicate the time (hours) relative to when the first sample was harvested and treatments began.
