# Supplementary figures and images for: Dawn and Dusk Set States of the Circadian Oscillator in Sprouting Barley (Hordeum vulgare) Seedlings
Source: PLoS One. 2015 Jun 11;10(6):e0129781. doi: 10.1371/journal.pone.0129781 (PMC4465908; doi:10.1371/journal.pone.0129781)

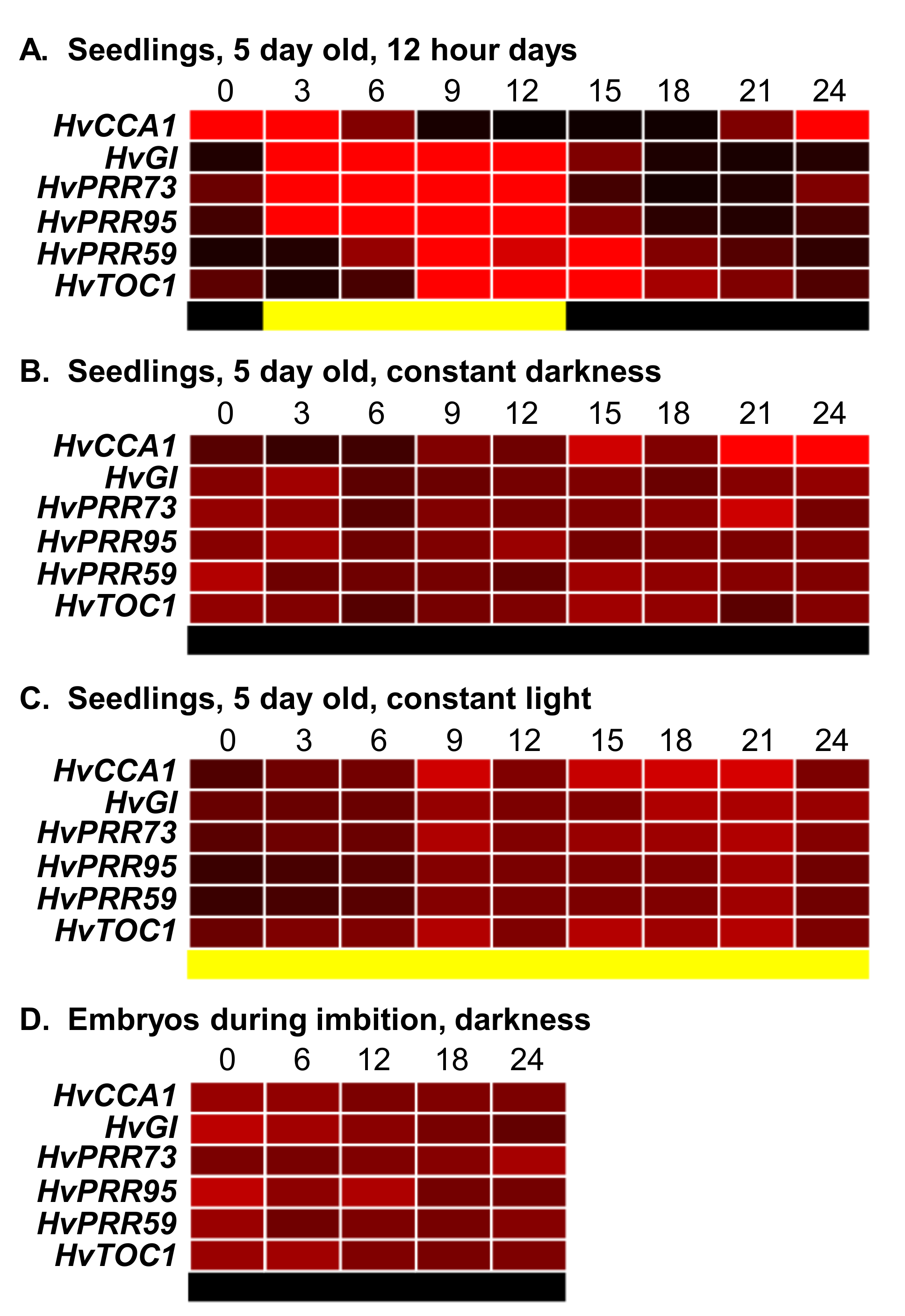

Supplement: S1 Fig — Heatmaps summarizing expression patterns of clock genes in barley embryos or 5 day old seedlings in different light regimes, at different timepoints (hours) after the start of each experiment. Average expression, assayed by qRT-PCR normalized to ACTIN (3 biological repeats), is presented ranged from 0 (black) to 2-fold increase (red) relative to median expression of that gene across all timepoints in the specific experiment. Horizontal axis labels indicate the time (hours) relative to when the first sample was harvested. Coloured bars underneath each heatmap indicate light conditions for each experiment across the timepoints examined. (TIF) [file pone.0129781.s001.tif]

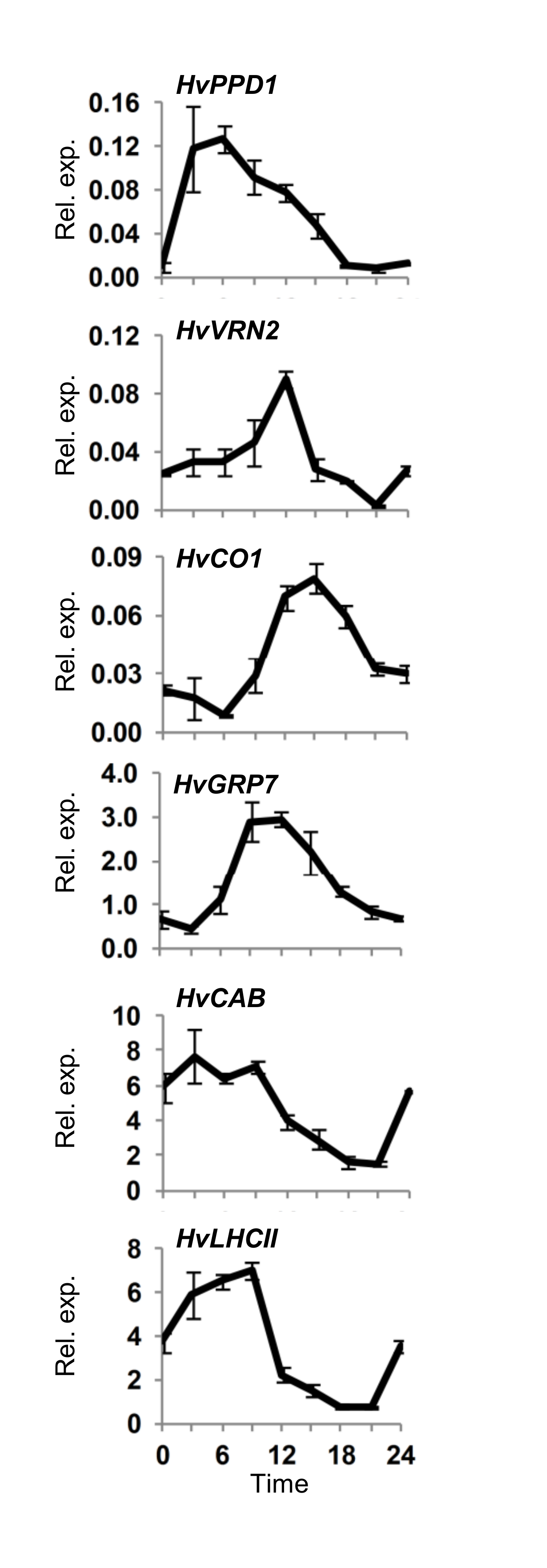

Supplement: S2 Fig — Gene expression patterns for clock-regulated genes, assayed by qRT-PCR, in 5 day old barley seedlings (cv. Sonja) germinated and grown in 12 hour day-night cycles. Average expression from 3 biological repeats is shown relative to ACTIN (Rel. exp.), error bars show standard error. Horizontal axis labels indicate the time (hours) relative to when the first sample was harvested. (TIF) [file pone.0129781.s002.tif]

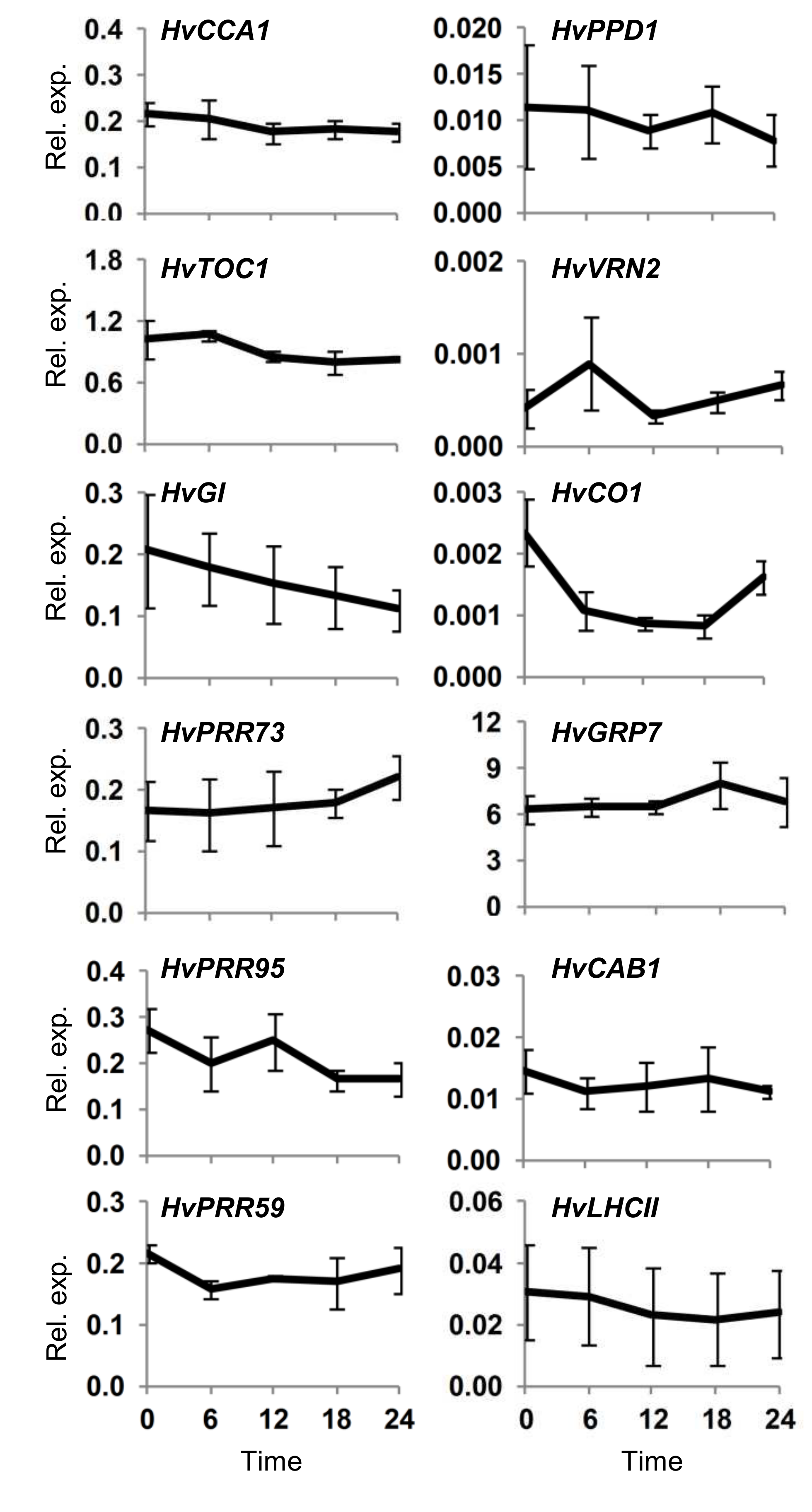

Supplement: S3 Fig — Gene expression, assayed by qRT-PCR, in barley embryos (cv. Betzes) isolated from dry seeds and at various timepoints after imbition. Detailed descriptions of morphological changes of embryos during the germination process during the time span have been presented previously by Barrero-Sanchez et al. [29]. Average expression is shown relative to GAPDH (Rel. exp.), error bars show standard error. Horizontal axis labels indicate the time (hours) relative to when the first sample was harvested. Similar results were obtained using ACTIN as a reference gene. (TIF) [file pone.0129781.s003.tif]

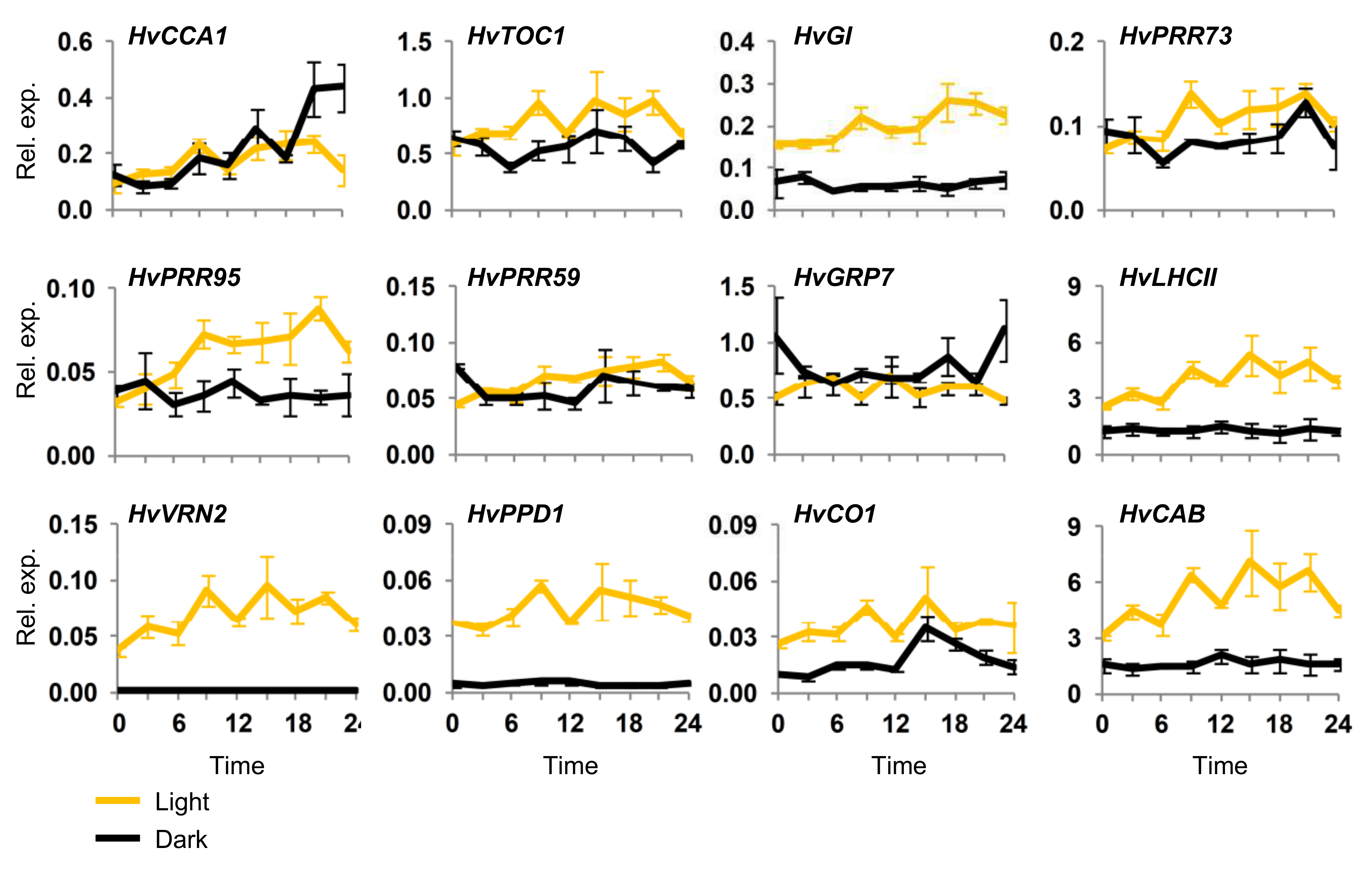

Supplement: S4 Fig — Gene expression, assayed by qRT-PCR, in 5 day old barley seedlings (cv. Sonja) that were germinated and grown in constant darkness (black) or constant light (yellow). Average expression (3 biological repeats) is shown relative to ACTIN (Rel. exp.), error bars show standard error. Horizontal axis labels indicate the time (hours) relative to when the first sample was harvested. (TIF) [file pone.0129781.s004.tif]

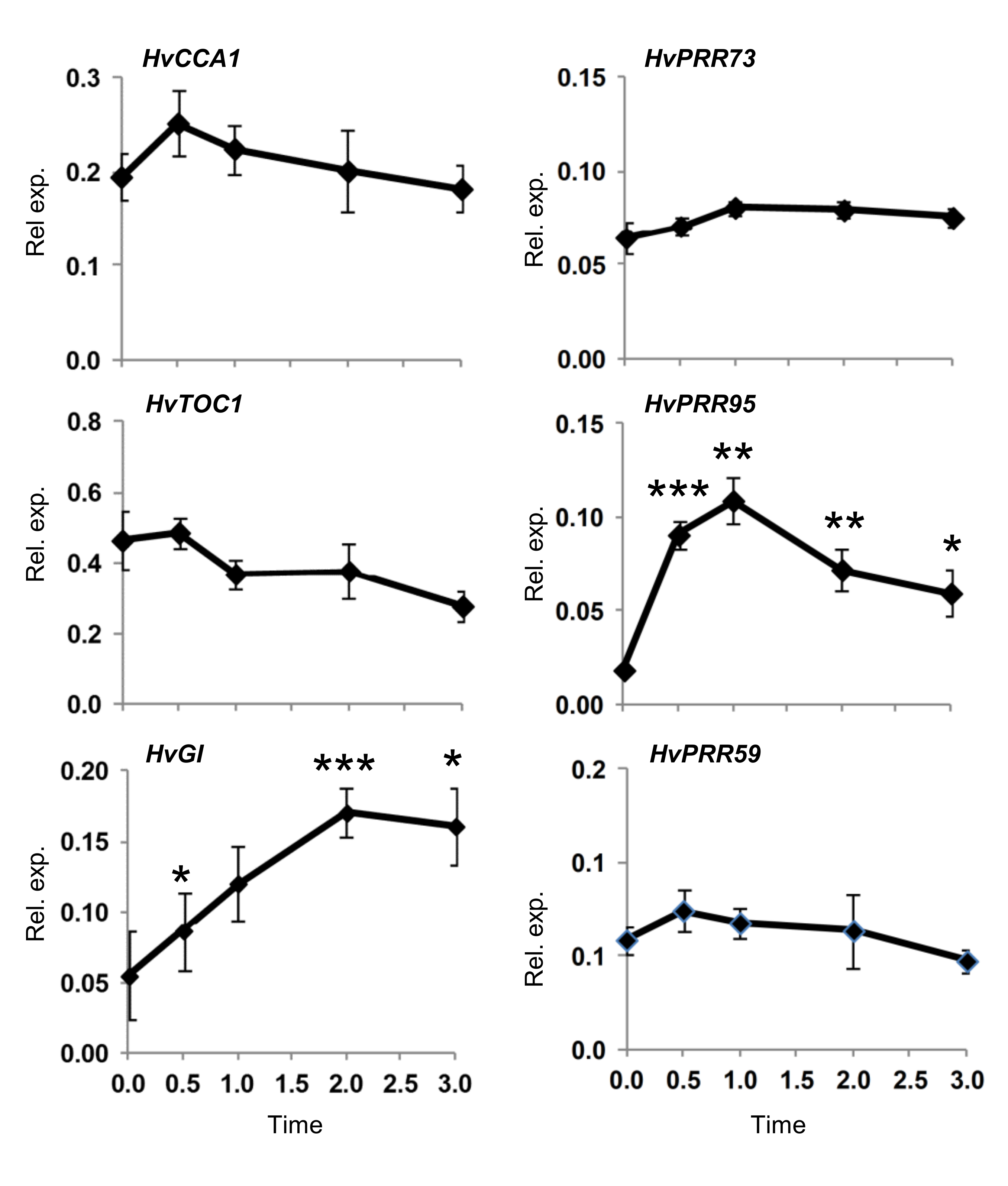

Supplement: S5 Fig — Barley seedlings (5 days old, cv. Sonja) were shifted from constant darkness to light and harvested at different timepoints from 0 to 3 hours. Gene expression was assayed by qRT-PCR. Data are presented for: HvCCA1, HvTOC1, HvGI, HvPRR73, HvPRR59 and HvPRR95. Expression is shown relative to ACTIN (Rel. exp.). Each data point is a mean of 3 biological repeats, error bars show standard error. Horizontal axis labels indicate time (hours) from the beginning of light exposure. * indicates Student’s T-test P<0.05, **P<0.01, ***P<0.001 versus the initial timepoint. (TIF) [file pone.0129781.s005.tif]

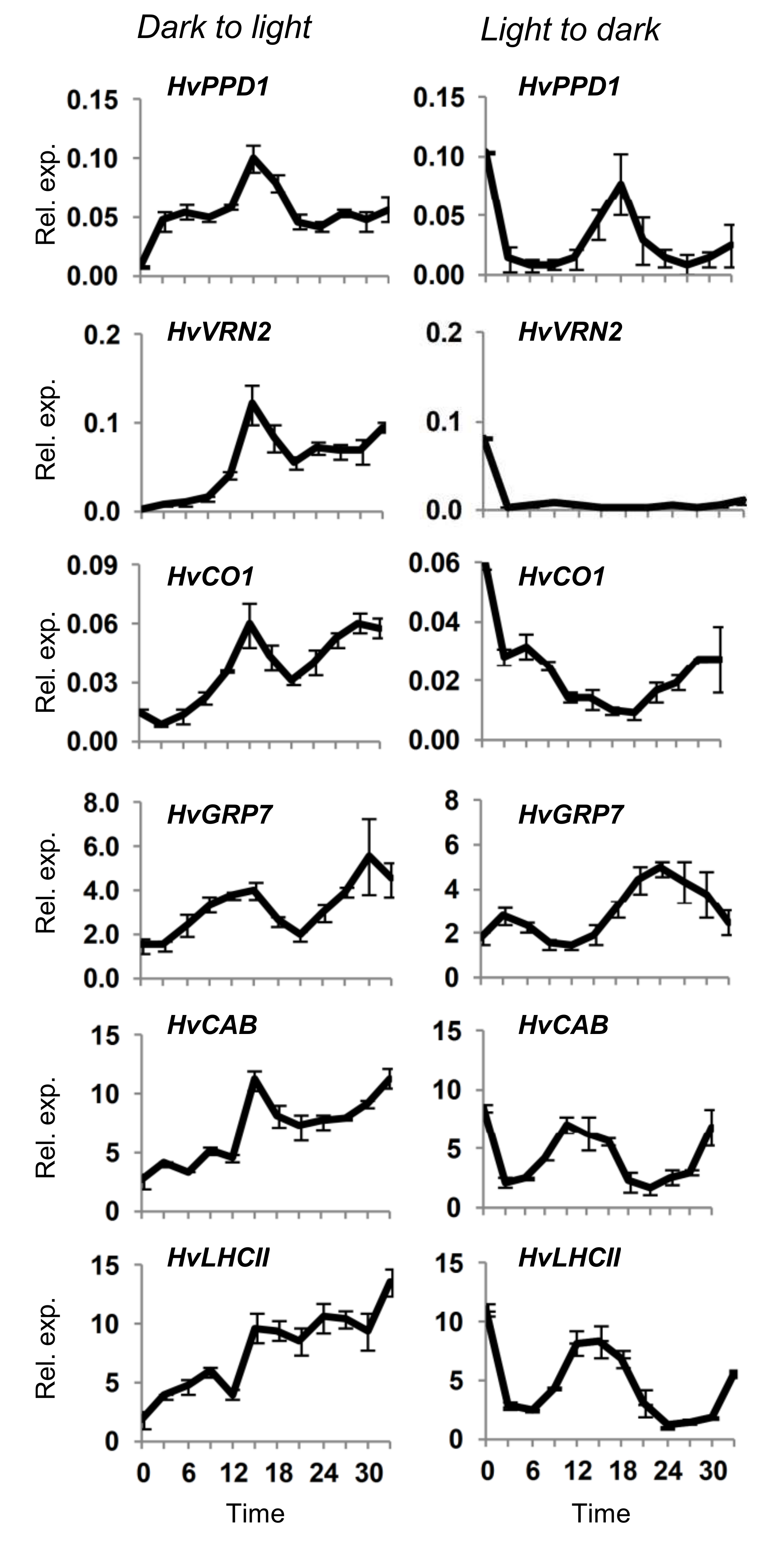

Supplement: S6 Fig — Gene expression, assayed by qRT-PCR, in 5 day old barley seedlings (cv. Sonja) germinated and grown in: constant darkness then shifted to light (left), or constant light the shifted to darkness (right). RNA was extracted from 3 biological repeats. Average expression is shown relative to ACTIN (Rel. exp.), error bars show standard error. Horizontal axis labels indicate the time (hours) relative to when the first sample was harvested and treatments began. (TIF) [file pone.0129781.s006.tif]

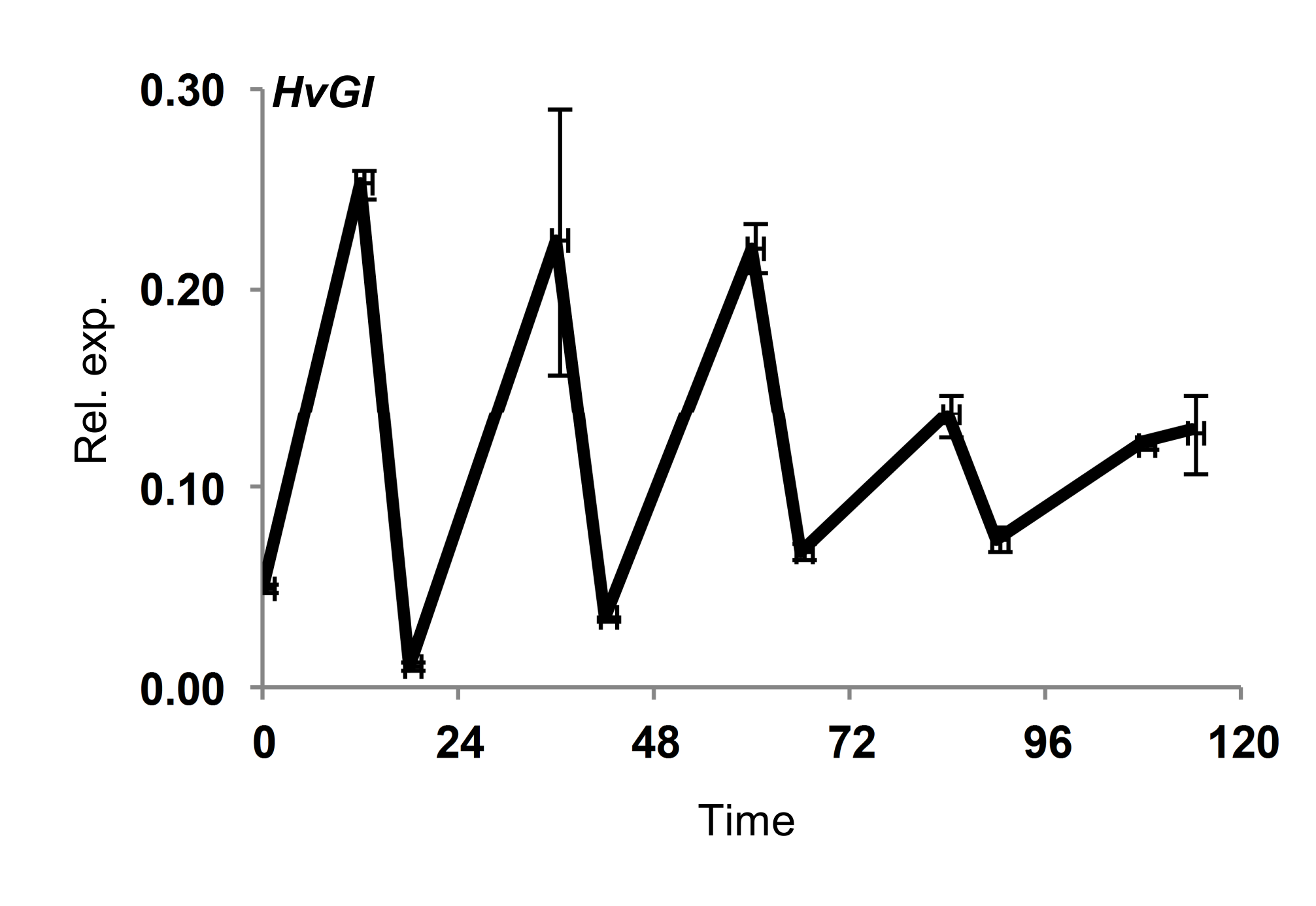

Supplement: S7 Fig — Barley seedlings (5 days old, cv. Sonja) were grown in constant darkness and then exposed to a 12 hour light pulse. Samples (2 biological repeats) were then collected at predicted times of peak (12 hours, then 36, 60 etc.) or trough expression (18 hours, 42, 66 etc), chosen on the basis of expression during the first 48 hours of the experiment, assayed at 3 hour intervals. Gene expression was assayed by qRT-PCR. Average expression is shown relative to ACTIN (Rel. exp.), error bars show range. Horizontal axis labels indicate the time (hours) relative to when the first sample was harvested. (TIF) [file pone.0129781.s007.tif]

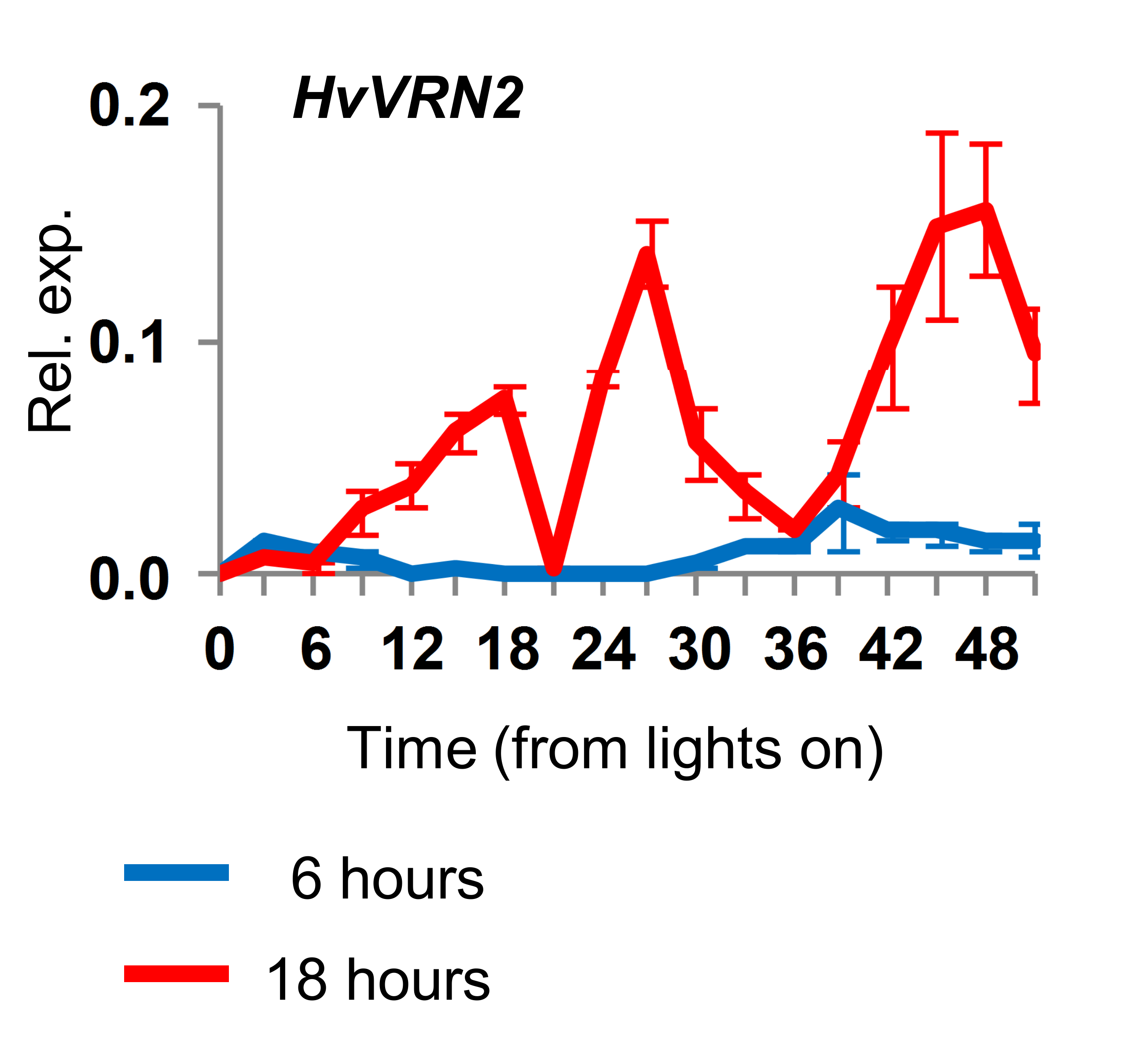

Supplement: S8 Fig — Average transcript levels of HvVRN2, relative to ACTIN, in barley seedlings grown in darkness and then exposed to a single light pulse of 6 (blue line) or 18 hours (red line). Expression (3 biological repeats) was assayed by qRT-PCR, error bars show standard error. Horizontal axis labels indicate the time (hours) relative to when the first sample was harvested. (TIF) [file pone.0129781.s008.tif]

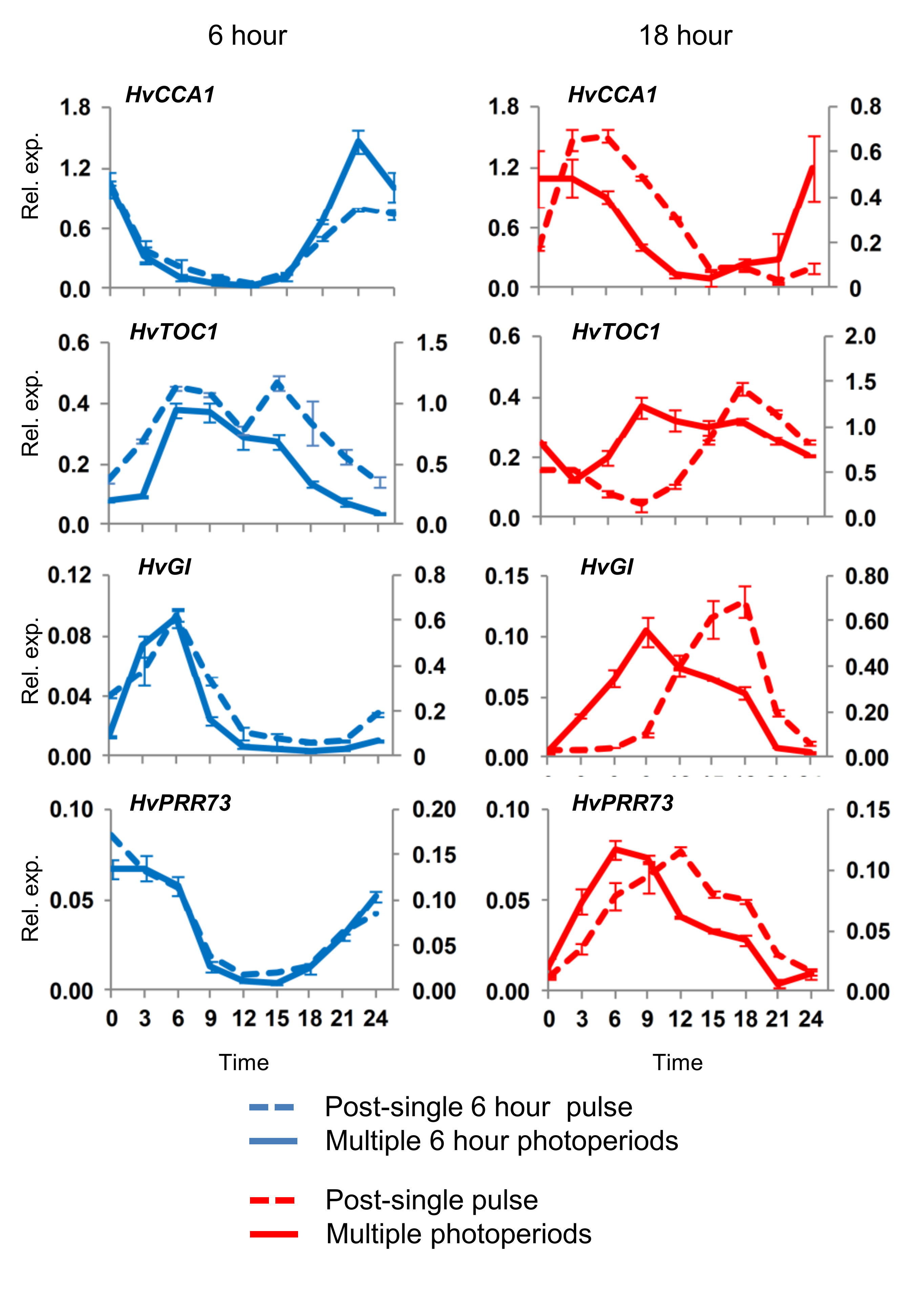

Supplement: S9 Fig — Clock gene expression in short (6 hour light) or long days (18 hours light) versus the second day after a single photoperiod pulse of 6 or 18 hours. RNA was extracted from 3 biological repeats. Average expression is shown relative to ACTIN (Rel. exp.), error bars show standard error. Horizontal axis labels indicate the time (hours) relative to when the first sample was harvested. The transcript levels of some genes vary between treatments and so are plotted on different scales (y-axis) to allow easier comparison of overall expression patterns (i.e. timing of oscillations). In each case, the multiple photoperiods treatment is plotted on the primary (left) axis, the single light pulse on the secondary (right-hand). (TIF) [file pone.0129781.s009.tif]

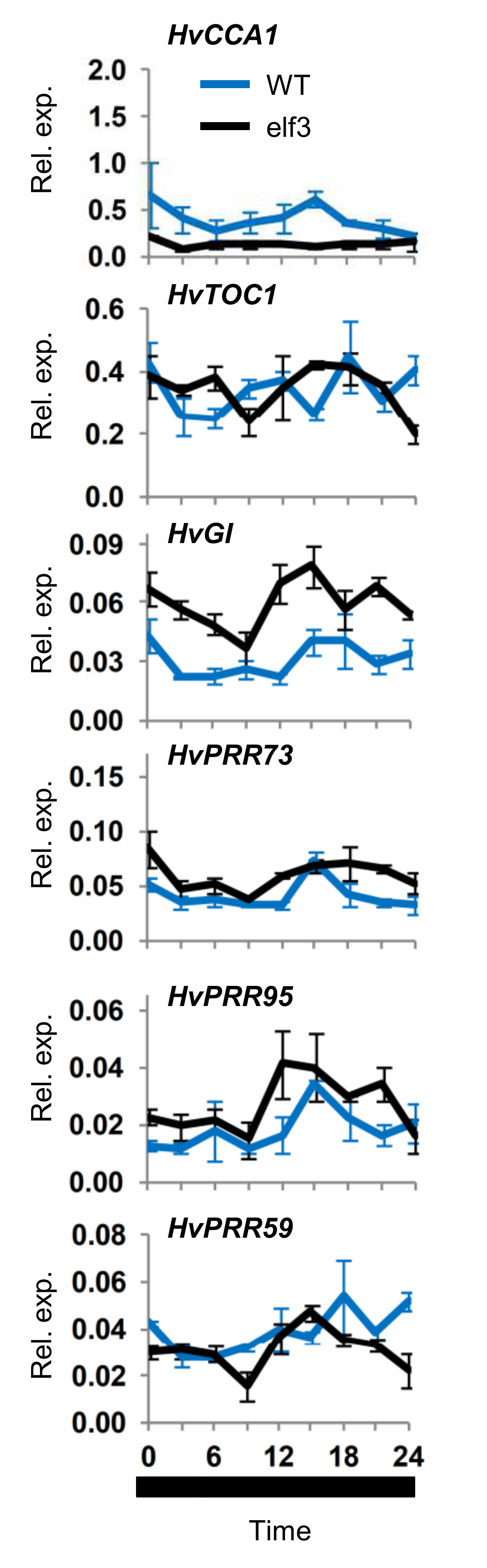

Supplement: S10 Fig — Transcript levels of clock genes assayed qRT-PCR and normalized to ACTIN (3 biological repeats) in 5 day old barley seedlings (cv. Bonus) that were germinated and grown in constant darkness compared to a HvELF3 loss-of-function mutant (black line). Error bars show standard error. (TIF) [file pone.0129781.s010.tif]

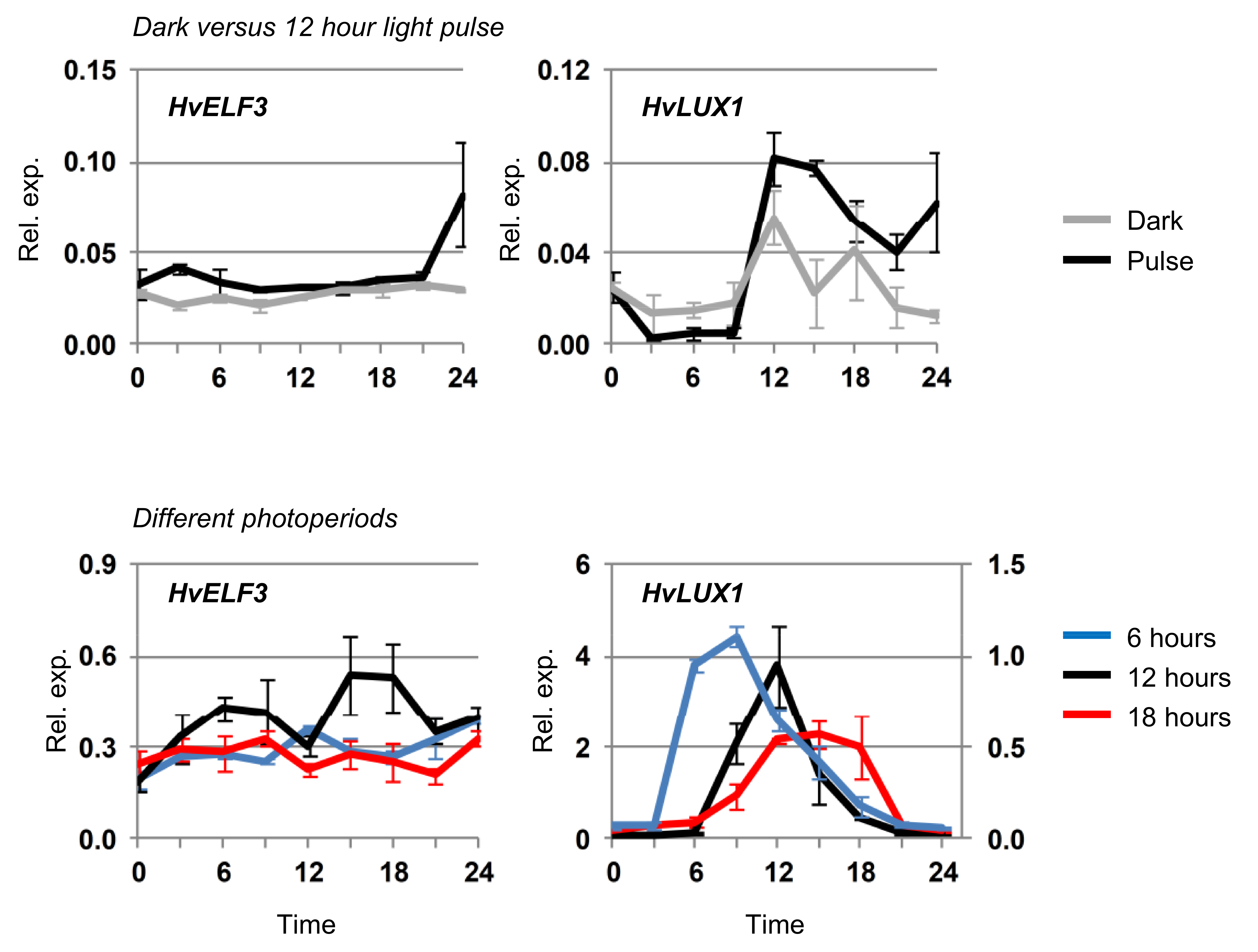

Supplement: S12 Fig — (A) Transcript levels of HvELF3 in 5 day old seedlings (cv. Sonja) grown in constant darkness versus constant darkness followed by a single 12 hour light treatment, which started on the 5th day. (B). Transcript levels of HvLUX1 in constant darkness versus single 12 hour light treatment. (C) Transcript levels of HvELF3 in 5 day old seedlings that were grown in 6, 12 or 18 hour photoperiods. (D) Transcript levels of HvLUX1 in 5 day old seedlings that were grown in 6, 12 or 18 hour photoperiods. The 6 hour photoperiod is plotted on the secondary axis. Expression was assayed by qRT-PCR. Average expression is shown relative to ACTIN (Rel. exp.), error bars show standard error. Horizontal axis labels indicate the time (hours) relative to when the first sample was harvested. (TIF) [file pone.0129781.s012.tif]
