# Supplementary material for: Dawn and Dusk Set States of the Circadian Oscillator in Sprouting Barley (Hordeum vulgare) Seedlings
Source: PLoS One. 2015 Jun 11;10(6):e0129781. doi: 10.1371/journal.pone.0129781 (PMC4465908; doi:10.1371/journal.pone.0129781)
Supplement: S1 Table — (DOCX) [file pone.0129781.s013.docx]

**Table S1 Primers for gene expression assays.**

| **Gene** | **Direction** | **Sequence (5’-3’)** | **Reference** |
| --- | --- | --- | --- |
| *ACTIN* | F | GCCGTGCTTTCCCTCTATG | Trevaskis et al., 2006 |
|  | R | GCTTCTCCTTGATGTCCCTTA |  |
| *HvCCA1* | F | CGACAAGACACAGCAAGCAT | This study |
|  | R | CTTCATCTTGCTCCCCTCTG |  |
| *HvGI* | F | AGGCGAAATGGTAATGTTGC | This study |
|  | R | CAGACATCTGCGTTTCAGGA |  |
| *HvPRR73* | F | GCAACATTTCGGGGAAGCTG | This study |
|  | R | TGCCATTTGAGCCCTGCTTT |  |
| *HvPRR95* | F | TGCACGTTGAAATCCCCTCA | This study |
|  | R | GATGCAACCCCTCCATGCTT |  |
| *HvPRR59* | F | AGTGGGGTTTTGCCCTCACA | This study |
|  | R | GCAGCACCAACAGGAATTGG |  |
| *HvTOC1* | F | TCCAGGGACGTTGAGTTGGTT | This study |
|  | R | TTTTGAGCGGTTGGGGGTTG |  |
| *HvGRP7* | F | ACTGGAGGGAGTGAATGGTG | Campoli et al., 2012a |
|  | R | GGAACGGTAGCGTCACATCT |  |
| *VRN2* | F | GAGCCACCATCGTGCCATTC | Trevaskis et al., 2006 |
|  | R | GCCGCTTCTTCCTCTTCTC |  |
| *HvCO1* | F | CGTGCTTCGGCATACGCCTTCC | Campoli et al., 2012b |
|  | R | CTGCTGGGGCTAGTGCTTAC |  |
| *HvPPD1* | F | GATGGATTCAAAGGCAAGGA | Campoli et al., 2012b |
|  | R | GAACAATTGGCTCCTCCAAA |  |
| *HvCAB1* | F | CCACCGACCCCGACCAGG | This study |
|  | R | CGGAGATGACGGTGAGCAGG |  |
| *HvLHCII* | F | TCTGAGGGTGGTCTCGATTA | Campoli et al., 2012b |
|  | R | CAACAAGACCCATGAGAAGG |  |
| *HvLUX1* | F | AATTCAGTCCACGGATGCTC | Campoli et al., 2013 |
|  | R | CTTCACTTCAGCTCCCCTTG |  |
| *HvELF3* | F | CCTACCGACAACAAGCAGAA | Zakhrabekova et al. 2012 |
|  | R | CATGAATTCCCCAGCTGTAG |  |
